# Stabilizing Crystal Framework of an Overlithiated Li_1+*x*_Mn_2_O_4_ Cathode by Heterointerfacial Epitaxial Strain for High-Performance Microbatteries

**DOI:** 10.1021/acsnano.3c08849

**Published:** 2023-12-13

**Authors:** Jie Zheng, Rui Xia, Sourav Baiju, Zixiong Sun, Payam Kaghazchi, Johan E ten Elshof, Gertjan Koster, Mark Huijben

**Affiliations:** † University of Twente, MESA+ Institute for Nanotechnology, P.O. Box 217, 7500AE Enschede, The Netherlands; ‡ Forschungszentrum Jülich GmbH, Institute of Energy and Climate Research, Materials Synthesis and Processing (IEK-1), Jülich 52425, Germany

**Keywords:** Epitaxial stabilization, Thin film, Overlithiated Li_1+*x*_Mn_2_O_4_, Spinel cathode, Jahn−Teller distortion, Lithium-ion microbatteries

## Abstract

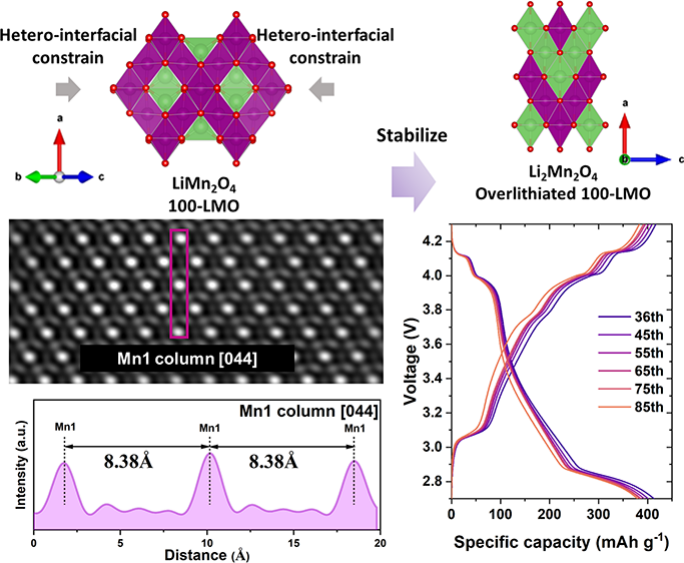

To meet the increasing demands of high-energy and high-power-density lithium-ion microbatteries, overlithiated Li_1+*x*_Mn_2_O_4_ (0 ≤ *x* ≤ 1) is an attractive cathode candidate due to the high theoretical capacity of 296 mAh g^–1^ and the interconnected lithium-ion diffusion pathways. However, overlithiation triggers the irreversible cubic-tetragonal phase transition due to Jahn–Teller distortion, causing rapid capacity degradation. In contrast to conventional lithium-ion batteries, microbatteries offer the opportunity to develop specific thin-film-based modification strategies. Here, heterointerfacial lattice strain is proposed to stabilize the spinel crystal framework of an overlithiated Li_1+*x*_Mn_2_O_4_ (LMO) cathode by epitaxial thin film growth on an underlying SrRuO_3_ (SRO) electronic conductor layer. It is demonstrated that the lattice misfit at the LMO/SRO heterointerface results in an in-plane epitaxial constraint in the full LMO film. This suppresses the lattice expansion during overlithiation that typically occurs in the in-plane direction. It is proposed by density functional theory modeling that the epitaxial constraint can accommodate the internal lattice stress originating from the cubic-tetragonal transition during overlithiation. As a result, a doubling of the capacity is achieved by reversibly intercalating a second lithium ion in a LiMn_2_O_4_ epitaxial cathode with a complete reversible phase transition. An impressive cycling stability can be obtained with reversible capacity retentions of above 90.3 and 77.4% for the 4 and 3 V range, respectively. This provides an effective strategy toward a stable overlithiated Li_1+*x*_Mn_2_O_4_ epitaxial cathode for high-performance microbatteries.

## Introduction

Energy storage plays a crucial role in realizing the energy transition in our society. Therefore, considerable effort is taken to achieve the next generation of lithium-ion batteries (LIBs) with higher energy/power densities, longer cycle life, and also low-cost and environmentally friendly processes.^1,2^ The cathode is of vital importance in realizing the promising performance of LIBs with satisfactory energy/power densities for practical applications.^3−5^ Of various kinds of known cathodes, the cobalt and nickel-free spinel-type LiMn_2_O_4_ (LMO) cathode spurred numerous studies due to the reversible Li^+^ insertion in the spinel framework at a suitable working potential (4.1 V vs Li) also in combination with the low toxicity and abundant source of Mn.^6,7^ In such a cubic spinel structure, the manganese ion occupies the octahedral site (16d), while the edge-sharing MnO_6_ octahedra provide a three-dimensional (3D) isotropic pathway through empty octahedral sites (16c) for a lithium ion that originally occupies the tetrahedral site (8a). Reversible Li^+^ intercalation in 8a sites leads to a theoretical capacity of 148 mAh g^–1^, although practically only 120∼130 mAh g^–1^ was achieved above the 4 V region, making LiMn_2_O_4_ fail to meet the growing demand of a higher energy density. Theoretically, beyond one lithium-ion insertion is possible for the octahedral vacancies (16c) in the spinel LMO framework when the voltage range is extended below 3 V to form the so-called overlithiated Li_1+*x*_Mn_2_O_4_ (0 ≤ *x* ≤ 1), which possesses a higher theoretical reversible capacity up to 296 mAh g^–1^.^8,9^ However, structural instability of overlithiated Li_1+*x*_Mn_2_O_4_ prevents its usability. The instability arises from the following two factors: (1) the increasing Mn^3+^ concentration in spinel LMO will inevitably lead to an irreversible phase transition from a cubic phase to a tetragonal phase because of a cooperative Jahn–Teller distortion;^9−11^ (2) Mn^2+^ that is generated by the disproportional reaction of Mn^3+^ will dissolve in the acidic electrolyte and cause an irreversible phase transformation at the surface.^6,12^ Thus, typically overlithiated Li_1+*x*_Mn_2_O_4_ results in a collapse of the crystal framework with a low energy/power density and poor cycling performance due to continuous degradation.

Various strategies have previously been proposed to stabilize the crystal structure of overlithiated Li_1+*x*_Mn_2_O_4_. For conventional LIBs, the bulk LMO particles could be stabilized by constructing surface modification layers,^13−15^ suppressing the cooperative Jahn–Teller distortion,^16,17^ and heavy alien element doping or embedding of inactive components.^18,19^ However, thin film lithium-ion microbatteries,^20^ which are developed to meet the tremendous demands of miniaturized electronic devices, offer specific thin-film-based modification strategies beyond the above-mentioned methods for conventional batteries. Although LMO thin film cathodes have previously been studied for microbatteries,^21−25^ stabilizing the spinel crystal structure of overlithiated Li_1+*x*_Mn_2_O_4_ thin film cathodes for microbatteries still remains a big challenge.

Epitaxial thin film growth on a single crystalline substrate allows the precise control of the crystal orientation of a material based on the substrate orientation and its interface structure adjacent to the underlying substrate. So far, several epitaxial LMO thin film model systems have been fabricated to elucidate valuable insight into the potential degradation mechanism in LMO.^26−28^ Moreover, (111) surface-dominated epitaxial LMO thin films with different crystal orientations have been investigated at the 4 V region to demonstrate enhanced high-rate electrochemical behavior and extended cycle life without excessive capacity fading.^29,30^ Recently, the low-temperature synthesis of epitaxial LMO films was enabled by the usage of a NiCo_2_O_4_ current collector.^31^ However, a stable, reversible overlithiation of an epitaxial LMO thin film cathode involving both 4 and 3 V regions is still lacking, which limits the implementation of thin film cathodes in high-performance microbatteries.

In this work, heterointerfacial lattice strain that originates from the lattice misfit at an epitaxial interface is proposed to stabilize the spinel crystal framework of overlithiated Li_1+*x*_Mn_2_O_4_. SrRuO_3_ (SRO) was introduced as a current collecting layer due to its good electronic conductivity, well-studied physical properties, and structural compatibility with the Nb-doped SrTiO_3_ (Nb:STO) substrates.^32−34^ Two distinct LiMn_2_O_4_ thin films were epitaxially deposited on top of the epitaxial SRO layers. Both types are distinct by their different out-of-plane crystal orientation of (100) and (110), denoted as 100-LMO and 110-LMO, respectively. As observed in high-angle annular dark-field scanning transmission electron microscopy (HAADF-STEM) and X-ray diffraction reciprocal space maps (RSMs), the LMO unit cell within the film interior exhibits a structural distortion with in-plane compression and out-of-plane elongation. The observed structural distortion indicates that the heterointerfacial lattice strain on the LMO film interior is an in-plane constraint. Detailed electrochemical analysis demonstrated stable and reversible Li^+^ (de)intercalation for both 100-LMO and 110-LMO films with reversible capacity retentions of above 90.3 and 77.4% at the 4 and 3 V regions, respectively. Distinct phase evolution after overlithiation has been studied by RSMs and complete reversible phase transition is proven, indicating the achieved stabilization effect of heterointerfacial lattice strain. Density functional theory (DFT) calculations demonstrated that the internal tensile lattice stress in Li_2_Mn_2_O_4_ can be mitigated by applying an external constraint. Furthermore, lithium kinetic analysis indicated that applying in-plane constraint would not compromise the lithium-ion diffusion in 100-LMO films. Consequently, heterointerfacial lattice strain has been demonstrated to stabilize overlithiated Li_1+*x*_Mn_2_O_4_ without the need for doping/embedding by heavy alien components. This provides an effective strategy to achieve a doubling of the capacity by reversibly intercalating a second lithium ion in an epitaxial LiMn_2_O_4_ cathode for high-performance thin film lithium-ion microbatteries.

## Results and Discussion

### Structural Characterization

**Figure 1:**
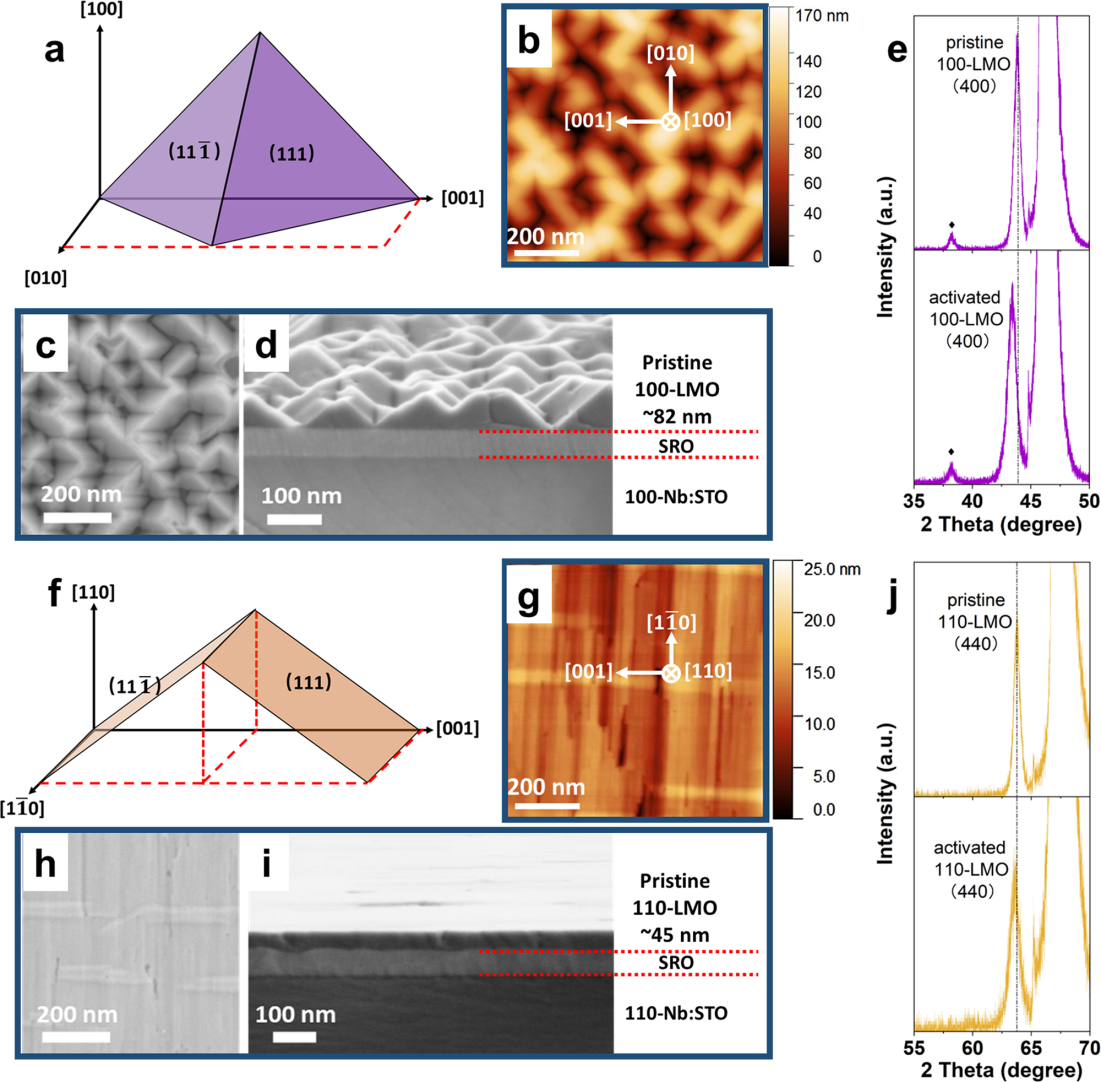
Schematics of surface morphologies of (a) 100-LMO and (f) 110-LMO films. AFM and SEM analysis of pristine (b–d) 100-LMO and (g–i) 110-LMO films. Zoomed-in XRD patterns with linear-scale *y*-axis of pristine and activated (e) 100-LMO films and (j) 110-LMO films. (c) and (h) correspond to the SEM top-view, while (d) and (i) display the cross-section view. The underlying Nb-doped (0.5 wt %) single crystalline SrTiO_3_ substrates are denoted as 100/110-Nb:STO. ⧫ in (e) indicates the minor contribution of the Mn_2_O_3_ impurity phase.

The epitaxial character of the LMO films was investigated by atomic force microscopy (AFM), scanning electron microscopy (SEM), and X-ray diffraction (XRD) analysis. It was reported that the surface energy decreases in the following order: {110} > {100} > {111}, among which {111} crystal facets are therefore energetically most favorable .^35,36^ This would imply that the formation of the surface crystal facet depends on temperature and reaction time.^36−38^ The morphology of epitaxial 100-LMO films is expected to exhibit a pyramidal structure when the {111} surfaces are exposed (Figure 1a). As shown in Figure 1b–d, the AFM and SEM images confirm the pyramidal character of pristine 100-LMO films,^29^ of which the truncated top is ∼45 ° rotated with respect to the [010] and [001] directions. It implies that under the applied growth conditions of 100-LMO films, the exposed surfaces are the {111} facets, which can effectively mitigate the dissolution of Mn ions.^28^ Additionally, the out-of-plane crystal alignment of the 100-LMO film with respect to the 100-Nb:STO was investigated by XRD, as shown in Figure S1a. The spinel LMO phase in pristine 100-LMO film presents a clear (400) peak, confirming the alignment of the pristine 100-LMO film along the [100] direction of the underlying 100-Nb:STO substrate. Figure 1e shows the zoomed-in XRD pattern, in which a small impurity phase of Mn_2_O_3_ is present because of the lithium volatility at the (100) plane of LiMn_2_O_4_.^39^ However, it will have a negligible effect on the electrochemical performance, as its redox potential is ∼1.3 V.^40^ X-ray photoelectron spectroscopy (XPS) was applied to study the surface elemental chemical properties of pristine 100-LMO films. Mn 2p XPS spectra shown in Figure S1b are composed of Mn^4+^ and Mn^3+^ peaks, and the ratio of Mn^4+^/Mn^3+^ is calculated to be 0.89, indicating that the average valance of Mn is +3.45 and thus confirming the negligible effect of the Mn_2_O_3_ impurity in the 100-LMO films. To activate the pristine 100-LMO film, CV measurement was conducted; see the experimental section. Two pairs of redox peaks between 3.8 and 4.3 V are observed in Figure S1c, indicating the typical (de)lithiation process through the 8a tetrahedral sites in the spinel LiMn_2_O_4_ structure. The (400) peak of the activated 100-LMO film, which is discharged to 3.6 V, shifts to the lower 2θ angle (Figure 1e), indicating the initial Li-poor state of pristine 100-LMO films and the compensation of Li^+^ in the well-aligned spinel framework after activation.

For the 110-LMO films, a rooftop-like morphology is expected to form when exposing the {111} facets (Figure 1f). The rooftop character for pristine 110-LMO is not clearly evident in the AFM and SEM images (Figure 1g–i) where surface morphology with elongated features is observed.^29^ It is attributed to the preferred diffusion of atoms along the [1̅10] and [11̅0] directions and the much less favorable diffusion along the [001] direction.^41^ However, the undulating rooftop character can be clearly seen in surface height profiles, as shown in Figure S2b. As comparison, pristine 100-LMO films exhibit much larger height differences (Figure S2a), which is attributed to the pyramidal structure exposing predominantly {111} facets. Because of the distinct morphology, 110-LMO films display a denser character with a reduced thickness compared to 100-LMO films (Figure 1d,i). Subsequently, the structure of the pristine 110-LMO films along the out-of-plane orientation was studied by XRD (Figure S3a and zoomed-in pattern in Figure 1j). The spinel LMO phase exhibits only a (440) peak, demonstrating the alignment of the pristine 110-LMO films along the [110] direction of the underlying 110-Nb:STO substrate. After electrochemical activation (Figure S3b), the out-of-plane orientation of the activated 110-LMO film is still properly aligned, except for a slight shifting of the (440) peak to lower 2θ values due to the compensation of Li^+^ after activation (Figure 1j).

**Figure 2:**
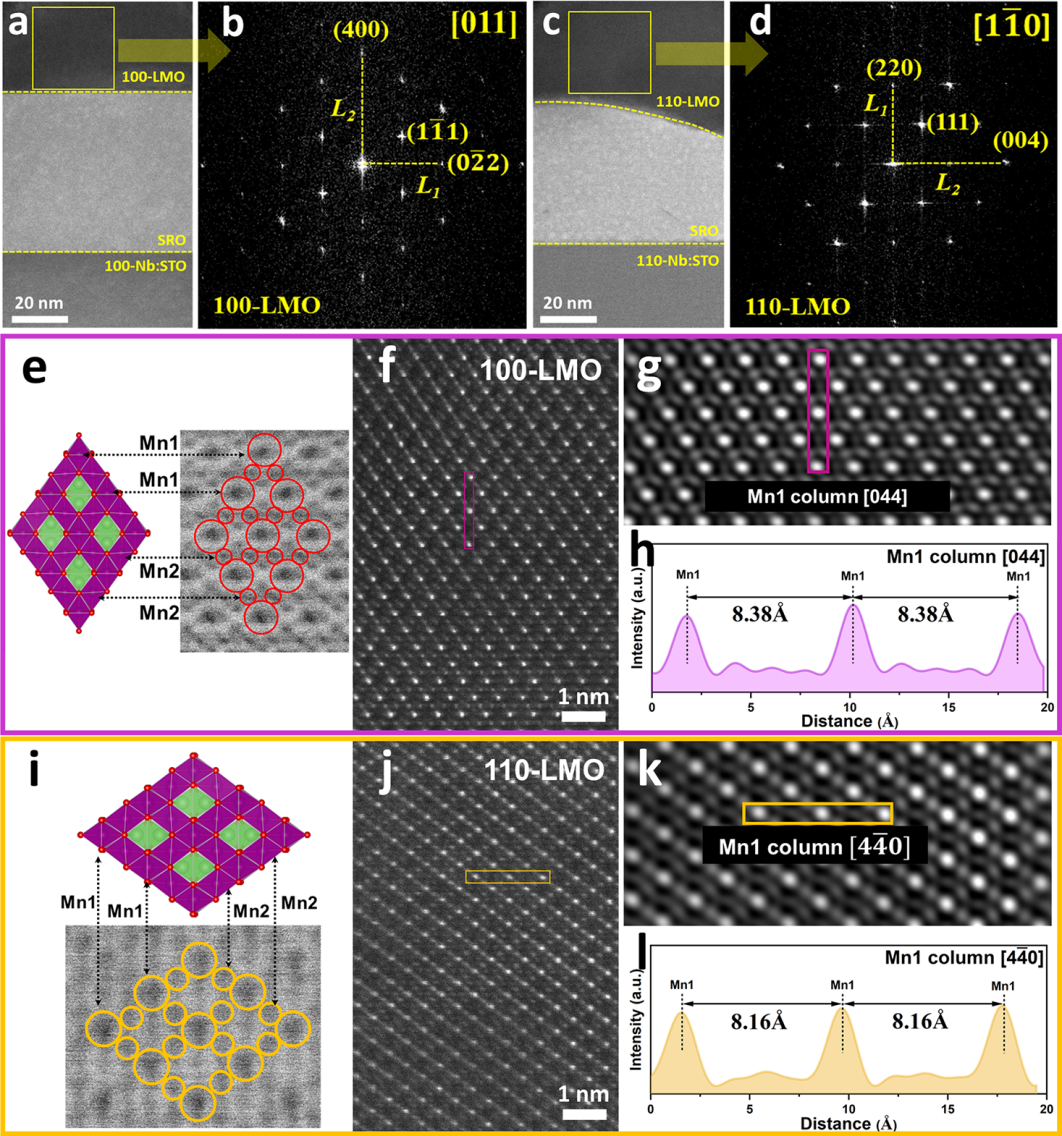
STEM images of epitaxial (a) 100-LMO/SRO/100-Nb:STO and (c) 110-LMO/SRO/110-Nb:STO configurations. The FFT images of (b) 100-LMO and (d) 110-LMO films correspond to yellow box areas in (a) and (c), respectively. Schematics of the LMO unit cell of (e) 100-LMO and (i) 110-LMO films. HAADF-STEM images of (f) 100-LMO and (j) 110-LMO film interiors. Inverse mask applied FFT of (g) 100-LMO and (k) 110-LMO films. Line profiles of the (h) Mn1 column along the [011] direction in (g) and the (l) Mn1 column along the [11̅0] direction in (k). Columns marked in (g) and (k) are the same as those marked in (f) and (j), respectively.

STEM imaging was applied to study in detail the atomic ordering within the spinel structures for different LMO films. Two lamellae were created by using the focused ion beam (FIB) technique along the [011] and [11̅0] zone axes for respectively activated 100-LMO and 110-LMO films, and their corresponding STEM images are shown in Figure 2a,c. Fast Fourier transform (FFT) analysis is performed on the yellow box regions, and the obtained FFT images of 100-LMO and 110-LMO films are shown in Figure 2b,d (raw images are shown in Figure S4), respectively. The FFT image of the 100-LMO film exhibits (400), (11̅1), and (02̅2) Miller planes, indicating the typical spinel structure. The FFT image of the 110-LMO film presents a similar spinel structure with (004), (111), and (220) Miller planes. The ratios of *L*_1_/*L*_2_, which correspond to *d*-spacing ratios of *d*(400)/*d*(02̅2) and *d*(004)/d(220), are determined to be 0.715 ± 0.007 and 0.696 ± 0.006 for 100-LMO and 110-LMO films, respectively. This ratio is 0.707 for bulk spinel LMO phase without strain.^42^ Therefore, it suggests the presence of structural distortions in spinel 100-LMO and 110-LMO films over a long-range due to strain by the heterointerface with the underlying SRO layer and Nb:STO substrate.

High-resolution STEM imaging was utilized to investigate the effect of epitaxial heterointerfacial lattice strain on the LMO crystal structural distortion. As shown in Figure 2e,i, annular bright field (ABF)-STEM images of the 100-LMO and 110-LMO films exhibit the atomic arrangements with a typical diamond configuration of the spinel structure, where the two distinct Mn sites are denoted as Mn1 and Mn2. Figure S5a,d shows the atomic arrangement of 100-LMO and 110-LMO films in the vicinity of the heterointerface with the underlying SRO layer. As for bulk spinel LMO, the lattice value is 8.248 Å.^42^*L*_LMO_ and *L*_SRO_ are applied to represent the distance of periodic atom columns, which corresponds to the sizes of one LMO unit cell and two SRO unit cells, respectively. Mn1 columns are the brightest because Mn (*Z* = 25) is heavier than Li (*Z* = 3) and O (*Z* = 8). For 100-LMO films, the corresponding intensity line profile of Mn1 columns is shown in Figure S5b, where the average *L*_*LMO*_, namely *L*_*LMO*_, is determined to be 11.51 Å, which is smaller than 11.66 Å of a bulk LMO unit cell (in-plane compression: 1.28%). Sr or Ru columns of the underlying SRO layer display a similar contrast due to the similarity of the atomic masses (*Z* = 38 and 44 for Sr and Ru, respectively), and the related intensity line profile is shown in Figure S5c. The average *L*_*SRO*_, namely *L*_*SRO*_, is calculated to be 11.06 Å, implying that the in-plane lattice parameter of two SRO unit cells along [010] or [001] is 7.82 Å. As all SRO layers exhibit a near-perfect epitaxial match to the underlying Nb:STO substrates (cubic of 3.905 Å), they exhibit very similar in-plane lattice parameters.

For 110-LMO films, *L*_*LMO*_ is measured to be 8.08 Å, corresponding to the in-plane lattice value of a single LMO unit cell near the heterointerface (Figure S5e). This in-plane *c* lattice value is smaller than 8.248 Å of a bulk LMO unit cell with a compression of 2.03%. The in-plane lattice value of the underlying two SRO unit cells is determined to be 7.81 Å by *L*_*SRO*_ in Figure S5f. The lattice misfit between bulk cubic LMO and SRO is ∼5.5%, which makes a full epitaxial match unlikely. However, the LMO unit cells near the interface in 100-LMO and 110-LMO films exhibit structural distortions with in-plane compressions of 1.28 and 2.03%, respectively, to accommodate the lattice misfit, thus enabling the epitaxial growth. The higher compression of the in-plane *c* lattice for 110-LMO films indicates that these films undergo more in-plane compressive strain than the 100-LMO films, which can be attributed to the different atomic arrangements of the (100) and (110) LMO planes. As shown in Figure S6a,b, the Mn columns align with the Sr columns on the (100) plane of LMO by 45° rotation to the in-plane *b* or *c* axis, through which the effect of lattice strain on *b* and *c* directions will be comprised. In contrast, the Mn columns on the (110) plane of LMO are compressed completely along the in-plane *c* direction (Figure S6c,d), leading to a more predominant compression of the in-plane *c* lattice value for 110-LMO films.

Besides the interface region, the interior part of the 100-LMO and 110-LMO films (Figure 2f,j), which was taken from the top half of the yellow box regions in Figure 2a,c, respectively, was characterized to investigate the trend of the structural distortion of the LMO unit cell. The mask applied to FFT images is transformed to inverse FFT images with higher contrast for accurate analysis (Figure S7). In detail, Figure 2g shows the inverse FFT image of a 100-LMO film, in which Mn1 columns are marked by the purple box to determine the out-of-plane *a* lattice parameter. The corresponding line profile shown in Figure 2h indicates that the measured *a* lattice value is 8.38 Å. The elongation along the *a* direction is accompanied with compressed in-plane *L*_*LMO*_ of 11.60 Å (Figure S7d,e). As for a 110-LMO film, laterally arranged Mn1 columns are marked by a yellow box in the inverse mask applied to FFT (Figure 2k) to measure the in-plane *c* lattice value. Figure 2 presents the line profile, which determines the *c* lattice value of 8.16 Å, implying the predominant in-plane compressive effect, which results in the elongation of the unit cell along the out-of-plane direction to 11.71 Å (Figure S7i,j). Therefore, the structural distortions of the unit cells in LMO films can be observed close to the heterointerface where it originates but also spreading within the film interior. From the heterointerface to the interior part, the in-plane *L*_*LMO*_ of a 100-LMO film changes from 11.51 to 11.60 Å, while that of the 110-LMO film changes from 8.08 to 8.16 Å. Although the results indicate the presence of a lattice strain gradient from the heterointerface to the interior of the LMO films, a significant distortion of the LMO unit cell from the relaxed and bulk structure is maintained.

**Figure 3:**
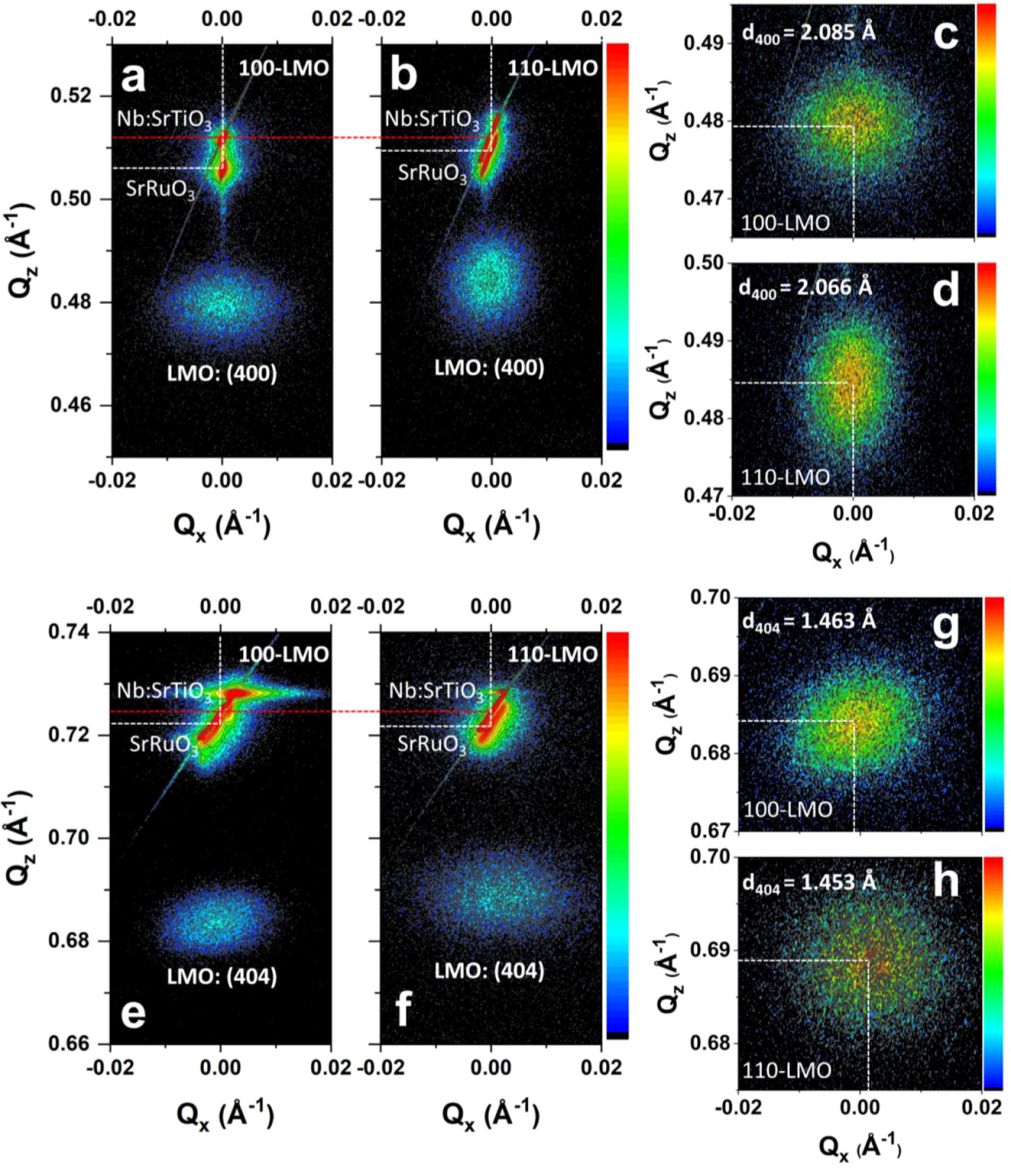
RSMs around the Nb:STO (200) peak for (a) 100-LMO and (b) 110-LMO films, and their corresponding zoomed-in LMO (400) peaks for (c) 100-LMO and (d) 110-LMO films. RSMs around the Nb:STO (202) peak for (e) 100-LMO and (g) 110-LMO films, and their corresponding zoomed-in LMO (404) peaks for (g) 100-LMO and (h) 110-LMO films.

To further investigate the structural distortions of the activated 100-LMO and 110-LMO films, 2θ–ω coupled XRD scans were performed to obtain RSMs around the (200) and (202) diffraction peaks of the Nb:STO substrates. As shown in Figure 3a, the 2D out-of-plane *Q*_*z*_*–Q*_*x*_ RSM of a 100-LMO film around the Nb:STO (200) peak exhibits the pronounced, narrow peaks of Nb:STO and SRO. Instead of leading to a Debye–Scherrer ring, the (400) plane of a 100-LMO film results in a well-defined peak with negligible mosaic tilt, indicating the good alignment of the LMO film along the [100] direction of the Nb:STO substrate. A similar conclusion can be drawn for the 110-LMO film based on the RSM around the Nb:STO (200) peak (Figure 3b). By changing the sample tilt, the RSMs of 100-LMO and 110-LMO films around the Nb:STO (202) peaks can be obtained as well (Figure 3e,f). The defined (404) peaks for both 100-LMO and 110-LMO films exhibit minimal mosaic twist with respect to the Nb:STO peaks, indicating the good alignment of both films along the [101] direction of the substrate as well as their single crystalline character. When the *Q*_*x*_ values are 0, the (200) and (202) Nb:STO peaks are located at the same *Q*_*z*_ position for both LMO films (marked with red dotted line). As the crystal structure of Nb:STO substrates remains constant, such a consistent *Q*_*z*_ can be used as a calibration of the peak position and indicates that the differences for the (400) and (404) LMO peak positions for 100-LMO and 110-LMO films should be attributed to significant structural distortions. In particular, the zoomed-in (400) LMO peaks reveal a *d*_400_ of 2.085 and 2.066 Å for 100-LMO and 110-LMO films, respectively (Figure 3c,d). On the other hand, the zoomed-in (404) LMO peaks indicate a *d*_404_ of 1.463 and 1.453 Å for 100-LMO and 110-LMO, respectively (Figure 3g,h). Therefore, the LMO lattice values can be determined (as illustrated in Figure S8) and are summarized in Table 1. Although the values determined by the RSM analysis are essentially averaged over the full LMO films, good agreement can be observed with the values determined by the STEM analysis from small regions in the interior of the films. Therefore, it is demonstrated that the in-plane compressive effect of the heterointerfacial lattice strain applies to the full LMO films, in which the LMO structure exhibits a structural distortion with an in-plane compression and an out-of-plane elongation.

**Table 1:** Lattice Values of the Spinel Crystal Structure for the Bulk LMO Unit Cell^42^ and the Activated LMO Films from STEM of the Film Interior and RSM Analysis of the Full Film

	Lattice values determined by STEM (Å)		Lattice values determined by RSM (Å)		
Axis	100-LMO	110-LMO	100-LMO	110-LMO	Bulk values (Å)^42^
a	8.38	/	8.340	8.264	8.248
b	/	/	8.213	8.264	8.248
c	/	8.16	8.213	8.175	8.248

### Electrochemical Analysis

**Figure 4:**
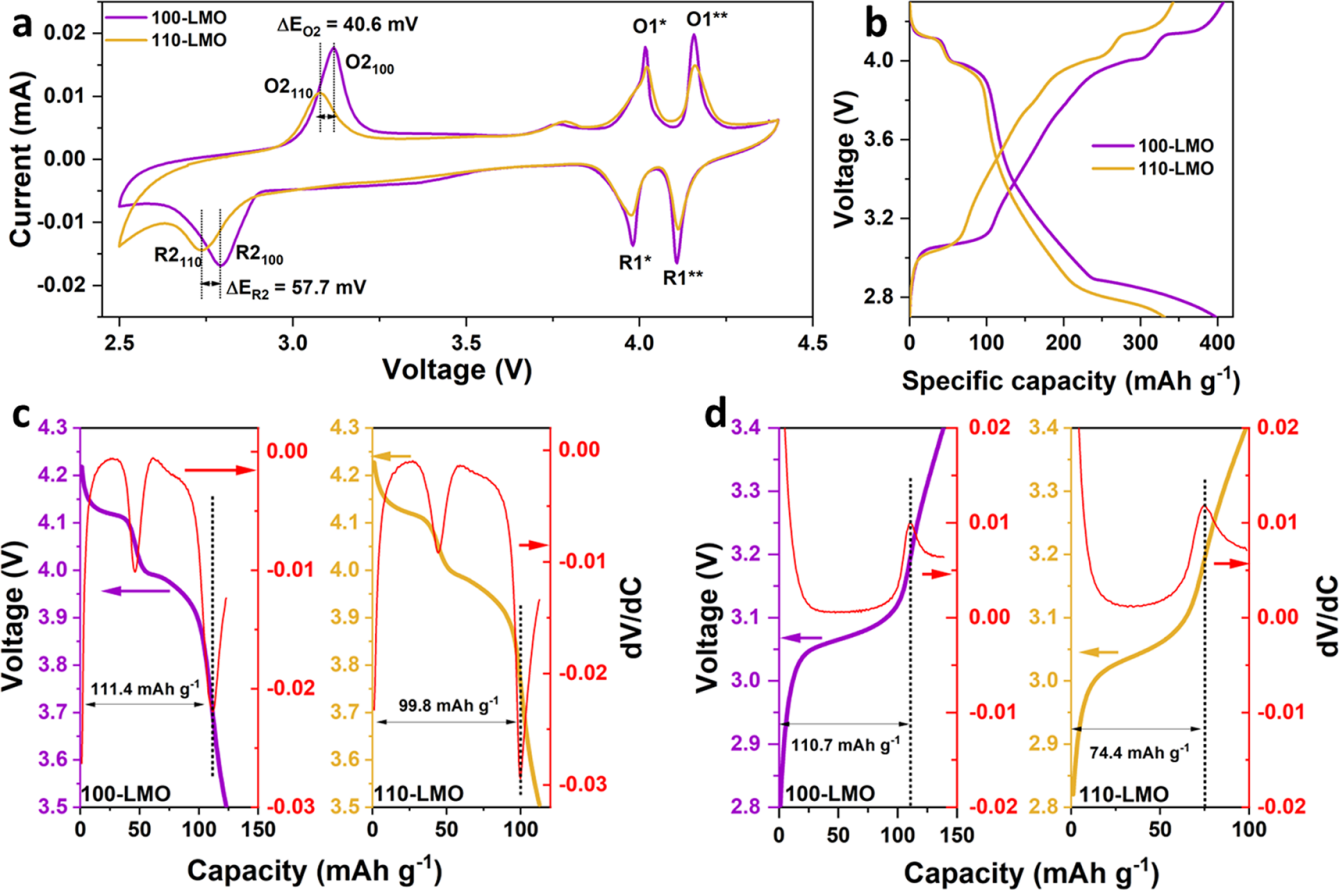
Electrochemical analysis of 100-LMO and 110-LMO films. (a) The CV profiles at the sweep rate of 1.0 mV s^–1^. (b) The charge/discharge curves at the current density of 40 μA cm^–2^. (c) The discharge curve and its first derivative profile in the voltage range of 3.5–4.3 V. (d) The charge curve and its first derivative profile in the voltage range of 2.8–3.4 V.

To study the electrochemical response of the structurally distorted LMO films, a series of electrochemical measurements, see experimental section, were applied. The redox behaviors of 100-LMO and 110-LMO films are investigated by cyclic voltammetry (CV) analysis (Figure 4a). Two pairs of well-defined redox couples (O1*/R1* and O1**/R1**) are observed at around 4 V for both types of LMO films, indicating the typical reversible (de)lithiation process in 8a tetrahedral sites in the spinel framework. Additionally, another pair of pronounced redox peaks (O2/R2) at ∼3.0 V corresponds to the overlithiation process in 16c octahedral sites leading to the phase transition from cubic LiMn_2_O_4_ to tetragonal Li_1+*x*_Mn_2_O_4_. All of these redox peaks are defined by the corresponding first-order derivative curves (Figures S9 and S10). Furthermore, the specific area of each defined peak, which is correlated to the Li^+^ content in the structure, is determined and is shown in Figure S11. In particular, the peak area ratio of *S*_O2–100_/*S*_O1–100_ is calculated to be ∼1.1 while that of *S*_R2–100_/*S*_R1–100_ is ∼0.94, suggesting a similar amount of (de)intercalated Li^+^ ions in the 3 V range compared to that in the 4 V range for 100-LMO films. However, *S*_O2–110_/*S*_O1–110_ and *S*_R2–110_/*S*_R1–110_ are ∼0.79 and ∼0.49, indicating less favorable Li^+^ intercalation in 16c octahedral sites for 110-LMO films. Additionally, the peak positions of O2_110_ and R2_110_ exhibit a shift to lower potentials with 40.6 and 57.7 mV as compared to O2_100_ and R2_100_, respectively, while the O1* and R1* peaks for both types of LMO films occur at very similar voltages with minimal difference (<5 mV, Figures S9 and S10). It is assumed that such a distinct difference in (de)lithiation behavior between 100-LMO and 110-LMO films for the 3 V range can be attributed to the different strain distributions, which will be discussed in the following section. Besides the main redox peaks, a tiny bump is also observed for both LMO films at ∼3.75 V and can be assigned to a Li^+^ deintercalation process in layered LiMnO_2_.^43,44^Figure 4b shows the charge/discharge curves at a current density of 40 μA cm^–2^ for the full 2.7–4.3 V range, where the plateaus can be clearly observed for both LMO films in good agreement with CV analysis. The capacity contribution from the potential range between the two plateau regions (middle region) is attributed to surface (side) reactions. To quantitatively verify these capacities, 100/110-LMO films with double thickness were fabricated, and the corresponding charge/discharge profiles are shown in Figure S12. The inflection points of the corresponding first-order derivative curves are applied to define middle regions and specific plateau regions, of which specific capacities can be extracted from the overall curves. As shown in Figure S12 and Table S1, the overall capacities of 100/110-LMO films scale with the film thickness, while the contribution of the middle region of thicker films remains similar to that of thinner films. These results demonstrate that the extra contribution from the middle region should be attributed to the surface (side) reaction rather than the faradaic component of the films. Regarding the plateau regions, as shown in Figures 4 and S13, a 100-LMO film delivers an overall charged capacity of 256.0 mAh g^–1^ (145.3 and 110.7 mAh g^–1^ at 4 V + 3 V) and discharged capacity of 225.3 mAh g^–1^ (111.4 and 113.9 mAh g^–1^ at 4 V + 3 V). As comparison, a 110-LMO film delivers a lower overall charged capacity of 209.2 mAh g^–1^ (134.4 and 74.4 mAh g^–1^ at 4 V + 3 V) and discharged capacity of 173.9 mAh g^–1^ (99.8 and 74.1 mAh g^–1^ at 4 V + 3 V). The lower reversible capacity of the 110-LMO film in comparison to the 100-LMO film for the 4 V range is in good agreement with previous studies^29^ and can be attributed to the lower surface area limiting the diffusion process. The underlying origin for the reduced capacity of a 110-LMO film for the 3 V region, which is seen in CV analysis as well, is discussed below in more detail.

**Figure 5:**
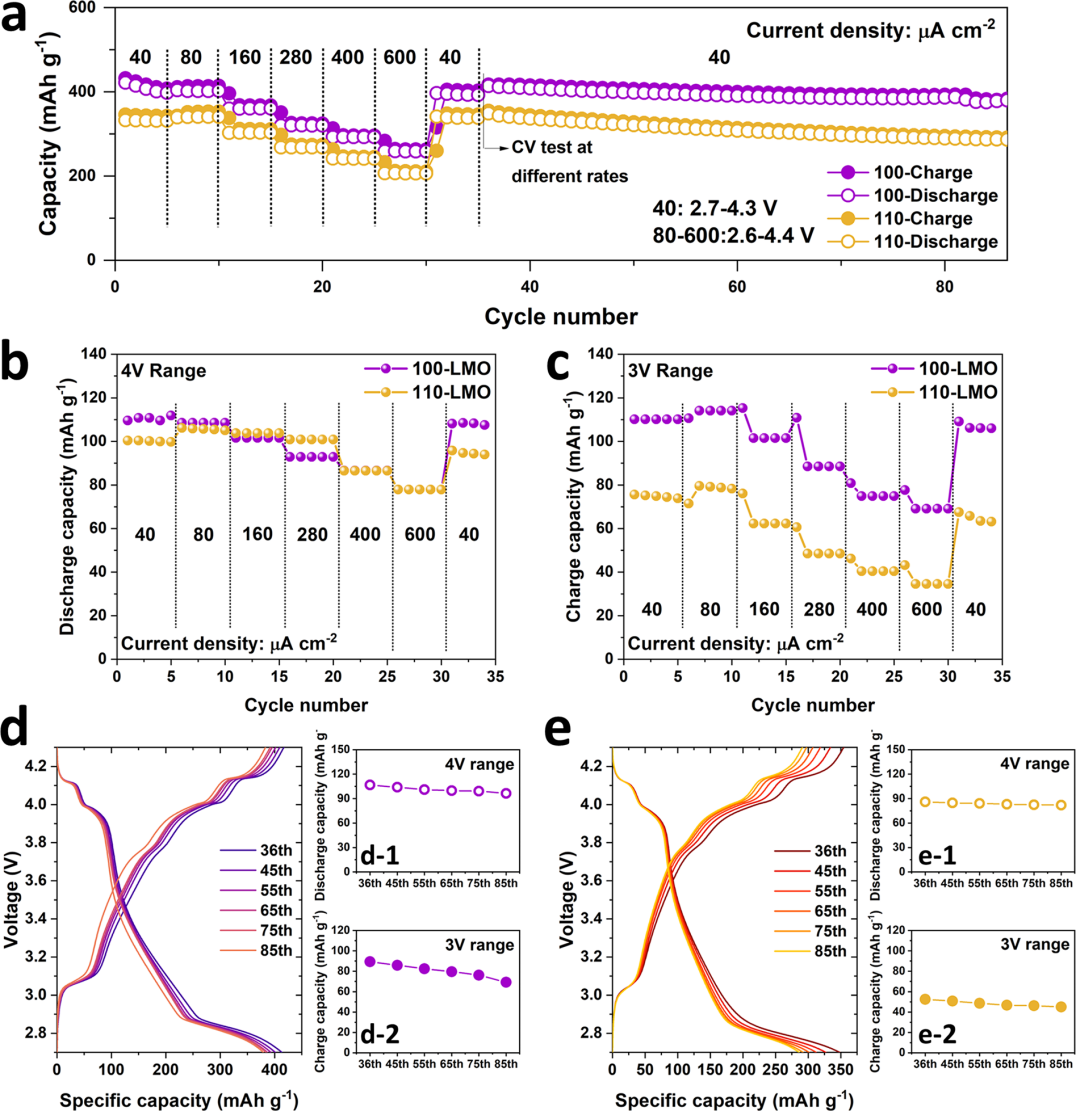
(a) Cycling performance of 100-LMO and 110-LMO films at various current densities. (b) Extracted discharge capacity at 4 V range. (c) Extracted charge capacity at 3 V range. Charge/discharge curves and corresponding extracted discharge or charge capacity of (d) 100-LMO and (e) 110-LMO films at different cycles.

To investigate the stability of the structurally distorted LiMn_2_O_4_ films, galvanostatic charge–discharge measurements at various current densities were performed. When a current of 40 μA cm^–2^ was applied, the potential range was limited to 2.7–4.3 V to prevent the influence of current leakage at relatively low/high voltages. Figure 5a shows that 100-LMO and 110-LMO films retain high stability at various current densities. To simplify the capacity extraction, discharge capacity at 4 V range and charge capacity at 3 V range are considered because they respectively indicate the reversibility of Li^+^ extraction from 8a tetrahedral sites (LiMn_2_O_4_ → Mn_2_O_4_ → LiMn_2_O_4_) and Li^+^ intercalation in 16c octahedral sites (LiMn_2_O_4_ → Li_2_Mn_2_O_4_ → LiMn_2_O_4_). As shown in Figure 5b, both LMO films exhibit similar rate performance for the 4 V range, whereas the 100-LMO film shows a better rate ability than the 110-LMO film for the 3 V range (Figure 5c). Subsequently, the LMO films were further cycled at a current density of 40 μA cm^–2^ for 50 cycles. The stable spinel structures of 100-LMO and 110-LMO films are demonstrated by high reversible capacity retentions of 90.3 and 95.3% for the 4 V range (Figure 5d-1,e-1). Regarding the 3 V range, reversible capacity retentions of 77.4 and 85.7% are achieved by 100-LMO and 110-LMO films, respectively (Figure 5d-2,e-2).

It is generally considered that nanosized materials can effectively accommodate the internal strain that originates from volume changes during cycling. A previous study by Put et al. found that LiMn_2_O_4_ thin film electrodes, though with nanosized thicknesses of 50 and 75 nm, show severe capacity degradation for the 4 V + 3 V region with less than 20% capacity retention after 30 cycles.^24^ Furthermore, Okubo et al. has proven the poor stability of nanocrystalline LiMn_2_O_4_ without domain boundaries.^45^ This indicates that nanosizing alone cannot effectively stabilize the spinel framework by accommodating the mechanical stress associated with the Jahn–Teller distortions. This suggests that the impressive stability of the 100/110-LMO films is strongly related to the heterointerfacial lattice strain and the corresponding structural distortion of the LMO spinel structure, instead of just downscaling the thin film layer thickness. Although reproducibility was demonstrated by numerous thin film samples, variations in results were observed due to possible contact issues between substrate and test device, as shown in Figure S14.

### Mechanism Investigation

**Figure 6:**
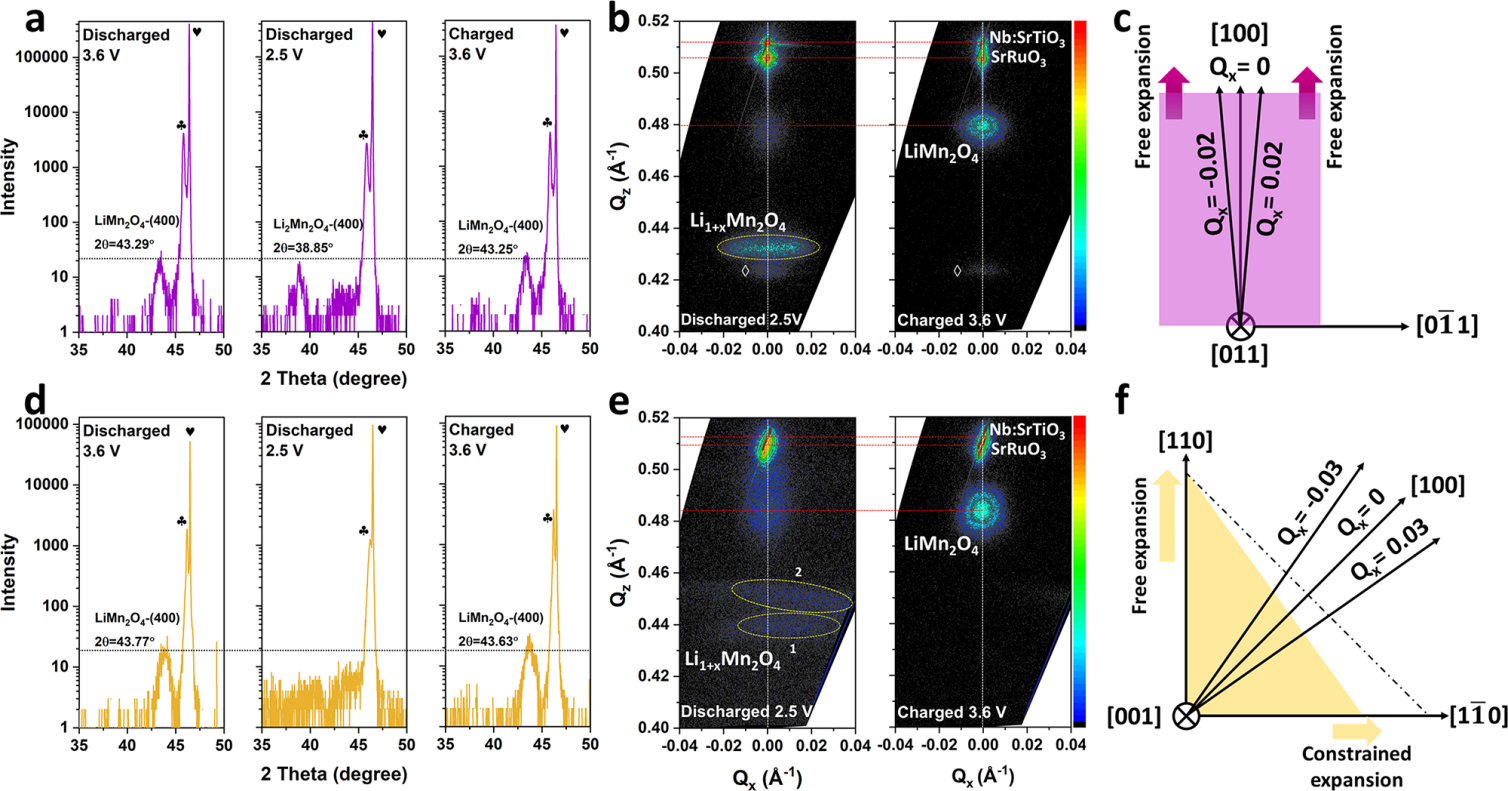
XRD patterns of (a) 100-LMO and (d) 110-LMO films at different lithiation states. RSMs of (b) 100-LMO and (e) 110-LMO films at different lithiation states around the Nb:STO (200) peak. Schematic illustration of lattice expansion behavior along the [100] direction in (c) 100-LMO and (f) 110-LMO films. ◊ in (b) indicates the tiny contribution from Mn_2_O_3_. XRD patterns in (a) and (d) were extracted in the case of omega without offset, which is corresponding to the *Q*_*x*_ position of 0 in RSMs.

To further understand the underlying mechanism that leads to the stable spinel structure and the distinct Li^+^ transfer kinetics in 100-LMO and 110-LMO films during the 3 V cycling, the structure evolution along the *a*-axis was investigated. As shown in Figure 6a, the (400) XRD peak of a 100-LMO film is clearly visible before overlithiation. When deeply discharged to 2.5 V, the (400) LMO peak shifts to a 2θ position of 38.85°, which can be indexed to the tetragonal Li_2_Mn_2_O_4_ phase,^9^ indicating a complete phase transition of cubic to tetragonal. The corresponding RSM shown in Figure 6b (Discharged 2.5V) displays the broadened peak of overlithiated Li_1+*x*_Mn_2_O_4_, which is symmetrical around the *Q*_*x*_ position of 0. As illustrated in Figure 6c, if the expansion along the *a*-direction is free of external strain, then the structure of the LMO unit cell should indeed be symmetrical around the *Q*_*x*_ position of 0. Therefore, this indicates that the lattice expansion of a 100-LMO film along the *a*-direction is not constrained. When charged back to 3.6 V, the LMO (400) peak shifts back to the original 2θ position of 43.25° with similar peak intensity as compared to the original (400) LMO peak before overlithiation (Charged 3.6 V vs Discharged 3.6 V in Figure 6a). The corresponding RSM (Charged 3.6 V in Figure 6b) shows a more well-defined and narrower (400) LMO peak as compared to LMO discharged to 2.5 V. Furthermore, the *d*_400_ of the 100-LMO film after being charged back to 3.6 V is determined to be 2.086 Å according to its magnified peak shown in Figure S15a. Based on above analysis, it can be concluded that the phase transition of the overlithiated 100-LMO film is completely reversible even though a full cubic-to-tetragonal phase transition occurs.

For the 110-LMO film, a significantly different behavior was observed for the crystal structure evolution during overlithiation. As shown in Figure 6d, the clearly defined (400) LMO peak of the 110-LMO film disappears when deeply discharged to 2.5 V, indicating that the initial well-aligned structure along the [110] direction is changed into a much less-ordered structure. Furthermore, the RSM of the 110-LMO film discharged to 2.5 V in Figure 6e shows the occurrence of two faint peaks in the *Q*_*z*_ range of 0.43–0.46 Å^–1^ (marked with areas 1 and 2). In particular, both peaks are not symmetric around the *Q*_*x*_ position of 0 and exhibit low intensity, indicating that the structure of the 110-LMO film has become disordered and no longer possesses significant long-range order after overlithiation. As illustrated in Figure 6f, the lattice expansion of the (400) LMO plane along the [100] direction should keep the symmetrical structure in case that expansion along the [110] and [11̅0] directions is free of compressive strain (indicated with dotted line). However, the symmetrical structure would be counteracted when the expansion along the [11̅0] axis is restricted by an external compressive strain (e.g., heterointerfacial lattice strain). Furthermore, a strain gradient would lead to an inhomogeneous expansion that breaks the ordered structure at the specific *Q*_*x*_ position. Thus, the evident asymmetry and weak intensity of both areas 1 and 2 suggest that the overlithiated-induced expansion of the (400) plane in 110-LMO films is constrained along the [11̅0] axis by the heterointerface lattice strain, which is expected to accommodate an internal lattice strain associated with Jahn–Teller distortions. When charged back to 3.6 V, the intensity of the (400) LMO peak is recovered, as can be observed in Figure 6d. Furthermore, its corresponding RSM (Charged 3.6 V in Figure 6e) exhibits again a well-defined (400) LMO peak, which suggests a *d*-spacing of 2.068 Å for the (400) LMO plane of the 110-LMO film (Figure S15b), indicating also here a completely reversible phase transition after overlithiation.

**Figure 7:**
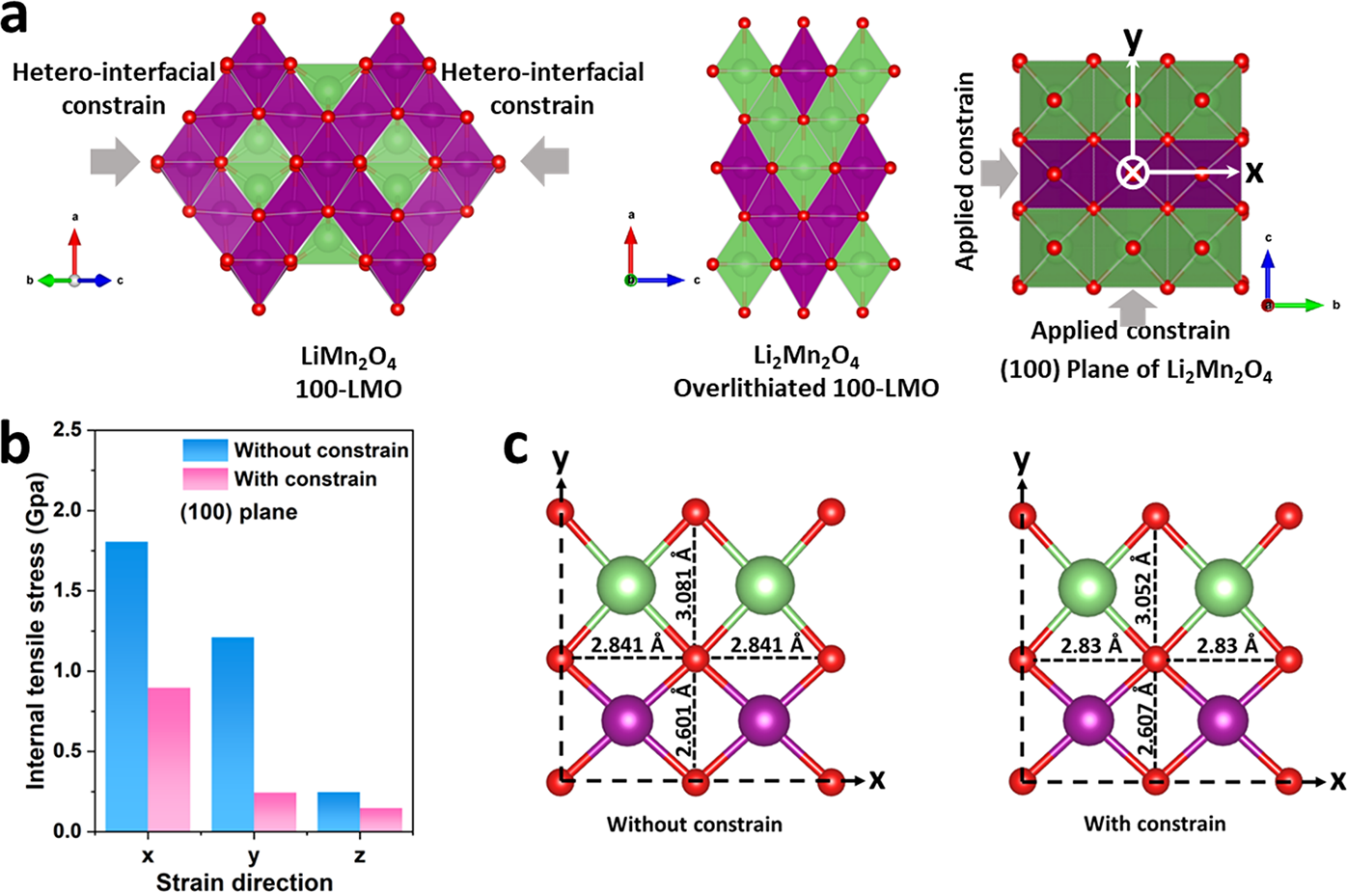
(a) Schematic structures of 100-LMO and overlithiated 100-LMO films, and the applied constrain on the (100) plane of Li_2_Mn_2_O_4_ for DFT modeling. (b) Internal lattice stress in the (100) plane of Li_2_Mn_2_O_4_ obtained by DFT calculation. (c) The *d*_*O-O*_ values in the (100) plane of overlithiated 100-LMO films obtained by DFT calculation.

The above results demonstrate that the heterointerfacial lattice strain suppresses the lattice expansions along the in-plane directions and exhibit negligible constrained effect on the lattice expansions along the out-of-plane direction. The lattice expansions of the 110-LMO films along all three axes (*a*, *b*, and c) are involving in-plane directions to stabilize the crystal framework. Interestingly, as the lattice expansion along the out-of-plane *a* direction for 100-LMO films is not constrained, they are still able to exhibit an impressive structural stability. The origin of stabilizing overlithiated 100-LMO films will be studied below in detail by DFT calculations. As illustrated in Figure 7a, the LMO unit cell in 100-LMO films is constrained by in-plane heterointerfacial strain along the [01̅1] and [011̅] directions, as demonstrated by STEM analysis (Figures 2, S5, and S7). After overlithiation, the tetragonal LMO phase is formed, and the in-plane constraints are along the *b* and *c* directions of the tetragonal unit cell. As RSM analysis reveals that overlithiated-induced expansion along the *a* direction is free of constraint, the (100) plane of the tetragonal Li_2_Mn_2_O_4_ unit cell is selected for DFT modeling. The simulated constraints are applied on the (100) slab model along the *x* and *y* directions, which are consistent with the in-plane *b* and *c* directions of the tetragonal Li_2_Mn_2_O_4_ unit cell. A constrain-free slab, for which the lattice parameters along *x* and *y* directions were fixed to corresponding fully optimized *b* and *c* lattice parameters of bulk model (Figure S16), was modeled as well. For both slab models, namely, without and with constraint from the heterointerface, the geometry along the *z* direction of the slab (i.e., *a* direction in bulk) was fully optimized. The DFT results shown in Figure 7b indicate that the (100) plane of the tetragonal Li_2_Mn_2_O_4_ unit cell is subjected to tensile stress. However, with the presence of applied external constraints, the internal tensile stress in the (100) plane is significantly reduced as compared to the situation without constraint. The O–O distances (**d*_O–O_*) were computed to explain the reduction of the lattice stress. As shown in Figure 7c, both without constraint and with constraint cases present the unchanging **d*_O–O_* values along the *x* direction. However, the difference of *d*_O–O_ values along the *y* direction is 0.480 Å for the model without constraint, which is larger than that for the model with constraint (0.445 Å) by 7.08%. Therefore, the system with constraint is more stable and hence experiences less stress. This result suggests that the heterointerfacial lattice strain not only suppresses the lattice expansion along the in-plane direction but also effectively accommodates the mechanical stress in the lattice along the out-of-plane direction, thus stabilizing the spinel framework during the overlithiation process.

### Lithium-Ion Transfer Kinetic Analysis

**Figure 8:**
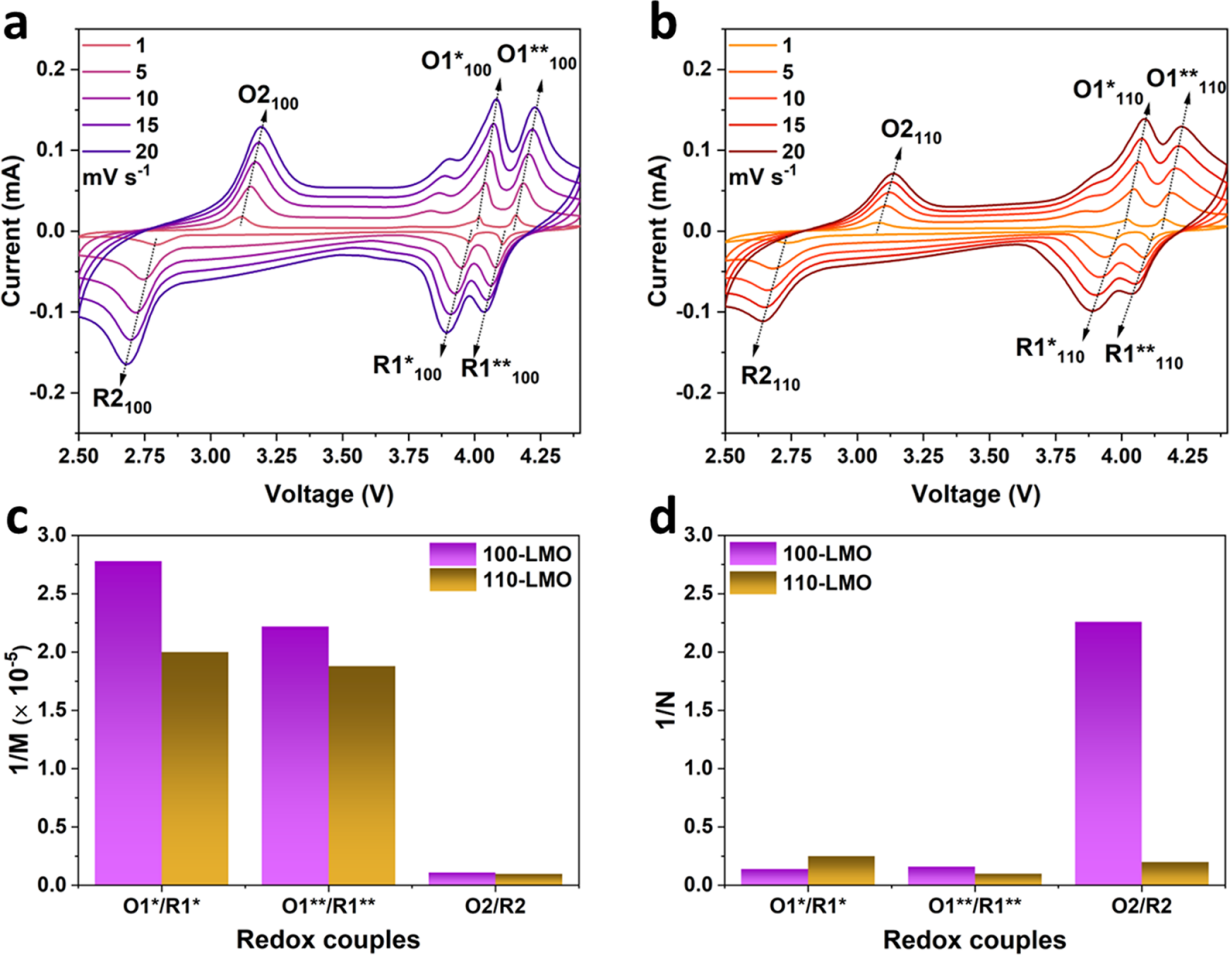
CV curves of (a) 100-LMO and (b) 110-LMO films at different sweep rates. (c) 1/*M* and (d) 1/*N* comparison for the different redox peaks of 100-LMO and 110-LMO films.

To get more insight into the distinct rate performance of overlithiated 100/110-LMO films, kinetic analysis based on CV curves at various sweep rates was performed. Figure 8a,b presents the CV curves of 100/110-LMO films at variable scan rates from 1 to 20 mV s^–1^. The redox peaks at 4 and 3 V regions are denoted as O1*/R1*, O1**/R1**, and O2/R2, respectively. Given the fact that the overlithiation involves a phase transition process, the impact of a sluggish electrochemical reaction rate should be taken into account. Therefore, a recently developed model is applied to separate the different current contributions of the charge and mass transfer processes, based on the following equations:^46^
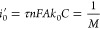
1
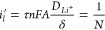
2
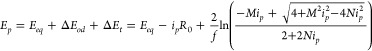
3where in eqs 1 and 2, *i*_0_^′^ and *i*_*l*_^′^ are the exchange current and the diffusion-limited current at maximum current, respectively. *A* is the effective area of the electrodes exposed to the electrolyte, while *n* represents the charge transfer number and *C* is the molar concentration of Li^+^ within the electrodes. In particular, *k*_0_ is the reaction rate constant and *D*_*Li*^+^_ is the diffusion coefficient. τ is the shape factor. *M* and *N* are defined as the elements to simplify eqs 1 and 2. In eq 3, *E*_*p*_ and *i*_*p*_ represent the voltage and the corresponding current at the current maximum, respectively. *E*_*eq*_ is the voltage at the equilibrium state. *ΔE*_*od*_ is the ohmic potential drop related to the ohmic resistance (*R*_0_). *ΔE*_*t*_ is the overpotential associated with the reaction rate constant (*k*_0_) and the diffusion constant (*D*_*Li*^+^_), thus with *M* and *N*. *ΔE*_*od*_ and *ΔE*_*t*_ result in the total potential drop Δ*E*. A series of *E*_*p*_–*i*_*p*_ points are collected to fit the curves of (*ΔE*_*od*_ + *ΔE*_*t*_)–*i*_*p*_ using eq 3. The fitted (*ΔE*_*od*_ + *ΔE*_*t*_)–*i*_*p*_ lines for O1*/R1*, O1**/R1**, and O2/R2 redox peaks are presented in Figure S17a–c, while the fitted components of *E*_*eq*_, *M*, and *N* are summarized in Table S2. In particular, 1/*M* and 1/*N*, which have a positive correlation with *k*_0_ and *D*_*Li*^+^_, respectively, are summarized in Figure 8c,d, respectively. It is observed that *1/N* is 4 to 5 orders of magnitude larger than *1/M*, implying that the (de)lithiation of Li_1+*x*_Mn_2_O_4_ is limited by the charge transfer process. However, all of the 1/*M* values for 100-LMO films are similar to those for 110-LMO films, suggesting that the charge transfer process is not a dominant origin for the observed variation of rate performance.

Interestingly, a significant variation of 1/*N* values for 100-LMO and 110-LMO films is observed. In detail, 1/*N* values for the O1*/R1* and the O1**/R1** of 100-LMO films are 0.14 and 0.16, respectively, which are close to those of 110-LMO films (0.25 and 0.10). As Li^+^ diffusion along [110] orientation is reported to be more favorable than [100] orientation,^36^ according to eq 2, these similar 1/*N* values suggest that the larger effective area (*A*) of 100-LMO films facilitates the mass transfer process at the 4 V region. However, 1/*N* values for the 1/*N* ratio of O2/R2 of 100-LMO films are 2.26, which is much higher than the 1/*N* value of 0.2 for 110-LMO films. Assuming that the exposed area of 100/110-LMO films remains unchanged during overlithiation, this dramatic difference indicates that the better rate performance of overlithiated 100-LMO films at the 3 V region might be mainly attributed to the more favorable Li^+^ diffusivity as compared to the 110-LMO films. As discussed before, the overlithiation-induced expansion of the (400) plane in 110-LMO films is suppressed by the heterointerfacial lattice strain, resulting in the disordered structure, which might make the Li^+^ diffusion and intercalation in 16c octahedral sites less favorable. As a comparison, the overlithiation-induced expansion of the (400) plane in 100-LMO films is free of constraint, maintaining the ordered tetragonal structure and thus allowing efficient Li^+^ diffusion at the 3 V range.

## Conclusion

Stabilization of epitaxial overlithiated 100/110-LMO films has been successfully achieved by heterointerfacial lattice strain. The structural distortion with an in-plane compression and out-of-plane elongation, as revealed by STEM and RSM analyses, suggests that the heterointerfacial lattice strain results in an in-plane constraint on the LMO unit cell. The structural distortion is observed over a long range within the epitaxial films, indicating the spread of strain from the interface area to the film interior region. With the presence of the heterointerfacial lattice strain, LMO films are able to achieve impressive cycling stability with reversible capacity retentions of above 90.3 and 77.4% for the 4 and 3 V ranges, respectively. Furthermore, 100-LMO films exhibit a better rate performance than 110-LMO films for the 3 V range while displaying a similar rate ability for the 4 V range. Structural evolution analysis demonstrates the completely reversible cubic-tetragonal phase transition for 100-LMO and 110-LMO films. The origin is revealed to be the compressive effect of the in-plane constraint on the overlithiation-induced lattice expansion, which is ascribed to the detrimental cubic-tetragonal transition. Furthermore, DFT calculations confirm that the in-plane constraint can also effectively mitigate the internal tensile stress within the plane, which expands along the out-of-plane direction. Lithium kinetic analysis based on CV measurements suggests that the better rate ability of 100-LMO films for the 3 V range can be attributed to the more efficient Li^+^ diffusion, which is caused by the conservation of a long-range ordered structure during the overlithiation process. This work provides an effective strategy to achieve a stable overlithiated Li_1+*x*_Mn_2_O_4_ epitaxial thin film cathode, which can be used for high-performance all solid state microbatteries by subsequently depositing a solid state electrolyte and thin film anode. Furthermore, this strategy can possibly be utilized to stabilize the spinel framework of overlithiated Li_1+*x*_Mn_2_O_4_ in particle-based batteries by constructing an epitaxial coating layer with a suitable bulk lattice misfit (e.g., ∼5.5% in this work) which is crucial to generate an effective epitaxial strain.

## Experimental Section

### Fabrication of LiMn_2_O_4_ Thin Film Electrode

First, the current collecting SrRuO_3_ (SRO) intermediate layer with a thickness of ∼60 nm was grown by pulsed laser deposition (PLD) on two types of Nb-doped (0.5 wt %) single crystalline SrTiO_3_ substrates (100/110-Nb:STO) from a sintered SrRuO_3_ target. Subsequently, LiMn_2_O_4_ (LMO) was grown on the obtained SRO layer from a sintered Li_2_Mn_2_O_4_ (100 wt % excess Li_2_O) target, to form LiMn_2_O_4_ thin films with different orientations (100 or 110-LMO). In detail, the oxygen partial pressure was 0.133 mbar, and the heater temperature was 635 °C during growth of both LMO and SRO layers. A KrF excimer laser operating at 248 nm was applied to generate a laser energy fluence of 2.3 J cm^–2^ with a frequency of 2 Hz. The applied pulses for all 100/110-LMO films were 7200. For 100/110-LMO films with double thickness, all parameters were the same, except 14 400 pulses were used. After deposition, the films were cooled to room temperature under an oxygen pressure of 0.133 mbar at 10 °C min^–1^.

### Materials Characterization

The crystal structure of the deposited LMO/SRO films was studied by XRD analysis (PANalytical X’Pert PRO diffractometer with Cu Kα radiation, λ = 0.15406 nm). Raw XRD data for RSMs were collected via multiple 2θ–ω coupled scans from a PANalytical- X’Pert material research diffractometer (MRD). The morphologies of the surfaces and the cross-sections of the films were characterized by AFM (Bruker ICON Dimension Microscope) and SEM (Zeiss Merlin HRSEM). The elemental surface composition was investigated by XPS (Omicron Nanotechnology GmbH surface analysis system with a photon energy of 1486.7 eV, Al Kα X-ray source). The atomic arrangements at the LMO/SRO heterointerfaces and within the LMO film interiors were investigated by HAADF-STEM (Thermo Scientific probe corrected Spectra 300). FIB (Thermo Scientific Helios 5 DualBeam) was utilized to create the LMO lamellae for STEM analysis. Lattice distance measurement and FFT were proceeded in DigitalMicrograph. The FFT images shown in Figure 2b,d were adjusted with brightness and contrast in DigitalMicrograph to display clearer detail. Their corresponding raw FFT images are shown in Figure S4a,b, respectively.

### Electrochemical Characterization

The obtained 100-LMO and 110-LMO thin films were studied as electrodes without conductive carbon or a polymer binder. The cells were assembled in an argon atmosphere glovebox and consisted of a LMO cathode, a lithium metal anode (99.9%, Sigma-Aldrich), and a glass fiber separator (ECC1-01-0012-B/L). 1.0 M LiPF_6_ dissolved in a 1:1 ratio v/v ethylene carbonate/dimethyl carbonate (EC/DMC, Sigma-Aldrich) was applied as the electrolyte. All electrochemical characterizations were conducted at room temperature in a galvanostat/potentiostat (VMP-300, Biologic) with EC-Lab software using EL-cells (ECC-ref). The electrochemical tests started with an activation process of the pristine LMO films via CV measurements at 1.0 mV s^–1^ from the open circuit voltage (OCV) to 4.3 V, followed by two additional CV tests between 3.6 and 4.3 V, leading to activated 100-LMO and 110-LMO films. Subsequently, CV measurements were performed at 1.0 mV s^–1^ in an extended potential range of 2.5∼4.4 V to study the full redox behavior of the 100- and 110-LMO films. Afterward, galvanostatic charge–discharge cycling was performed at various current densities (40, 80, 160, 280, 400, 600 μA cm^–2^) between 2.6 and 4.4 V to investigate the rate performance. The subsequent CV tests between 2.5 and 4.4 V were conducted under different sweep rates (1, 5, 10, 15, and 20 mV s^–1^) to enable detailed kinetics analysis. Finally, the cycling stability was analyzed by galvanostatic charge–discharge cycling at 40 μA cm^–2^ between 2.7 and 4.3 V. For the RSM measurements, the activated 100-LMO and 110-LMO films were cycled at a potential range of 2.5∼4.4 V for five cycles by CV scan at 1.0 mV s^–1^. The 100/110-films at “discharged 2.5 V” and “charged 3.6 V” were prepared by stopping the reduction scan at 2.5 V and the oxidation scan at 3.6 V, respectively, after five cycles. The calculations of the gravimetric capacities are based on eqs 4 and 5:^29−31^
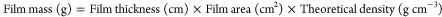
4
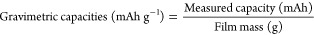
5

Given the fact that all 100/110-films were deposited using the exact same number of PLD pulses (7200 pulses), the amount of obtained LMO unit cells is the same for all films. Therefore, the thickness of the dense 110-LMO film was used to calculate the film mass for both the 100-LMO and 110-LMO films. The film area is the size of the substrate (5 × 5 mm). Theoretical density of cubic LiMn_2_O_4_ is 4.28 g cm^–3^.^29−31^

### Theoretical Modeling

The spin polarized DFT calculations were performed using the projected augmented wave (PAW) method within the Vienna Ab-initio simulation package (VASP).^47^ The Perdew–Burke–Ernzerhof (PBE)^48^ functional with generalized gradient approximation (GGA)^49^ was chosen as the exchange correlation functional for all calculations. The (100) surface structure of Li_2_Mn_2_O_4_ was constructed by using the optimized geometry of its bulk. A vacuum size of 20 Å was applied for the slab modeling the surface to avoid interaction between periodic images. In order to model the applied constraint in the experiment, the *x* and *y* lattice parameters of slab were fixed to 5.66 Å (*b* and *c* directions of bulk). The normal stress values on each direction (*xx*, *yy*, and *zz*) were then calculated. For all DFT-PBE calculations, an electronic convergence criterion of 10^–4^ eV and a force convergence of 0.01 eV/Å were applied. In addition, an energy cutoff of 700 eV and k-point mesh of 2 × 2× 1 were used.

## Supporting Information Available

Materials characterization including XRD, XPS, AFM, STEM, FFT, and RSM analysis of 100/110-LMO films. Schematic illustration of the crystal structure of LMO and SRO. Schematic illustration of geometry relations in 100/110-LMO models. Electrochemical analysis by various models based on CV data and the corresponding fitted results. Additional DFT modeling results for bulk Li_2_Mn_2_O_4_ (PDF)
