# Supplementary material for: Stabilizing Crystal Framework of an Overlithiated Li1+xMn2O4 Cathode by Heterointerfacial Epitaxial Strain for High-Performance Microbatteries
Source: ACS Nano. 2023 Dec 13;17(24):25391–404. doi: 10.1021/acsnano.3c08849 (PMC10753873; doi:10.1021/acsnano.3c08849)
Supplement: Supplementary file 1 — nn3c08849_si_001.pdf [file nn3c08849_si_001.pdf]

Supporting Information

Stabilizing Crystal Framework of Overlithiated $Li_{1+x}Mn_2O_4$ Cathode by Hetero-interfacial Epitaxial Strain for High-Performance Microbatteries

[+]Jie Zheng[a], [+]Rui Xia[a], Sourav Baiju[b], Zixiong Sun[a], Payam Kaghazchi[a,b], Johan E ten Elshof[a],

Gertjan Koster[a] and Mark Huijben[*,a]*

[a] University of Twente, MESA+ Institute for Nanotechnology, P. O. Box 217 7500AE Enschede, the Netherlands.

[b] Forschungszentrum Jülich GmbH, Institute of Energy and Climate Research, Materials Synthesis and Processing (IEK-1)Jülich 52425, Germany.

[+]These authors contributed equally to this work.

*Corresponding authors: m.huijben@utwente.nl

Keywords: Epitaxial stabilization, Thin film, Overlithiated $Li_{1+x}Mn_2O_4$, Spinel cathode, Jahn-Teller distortion, Lithium-ion microbatteries

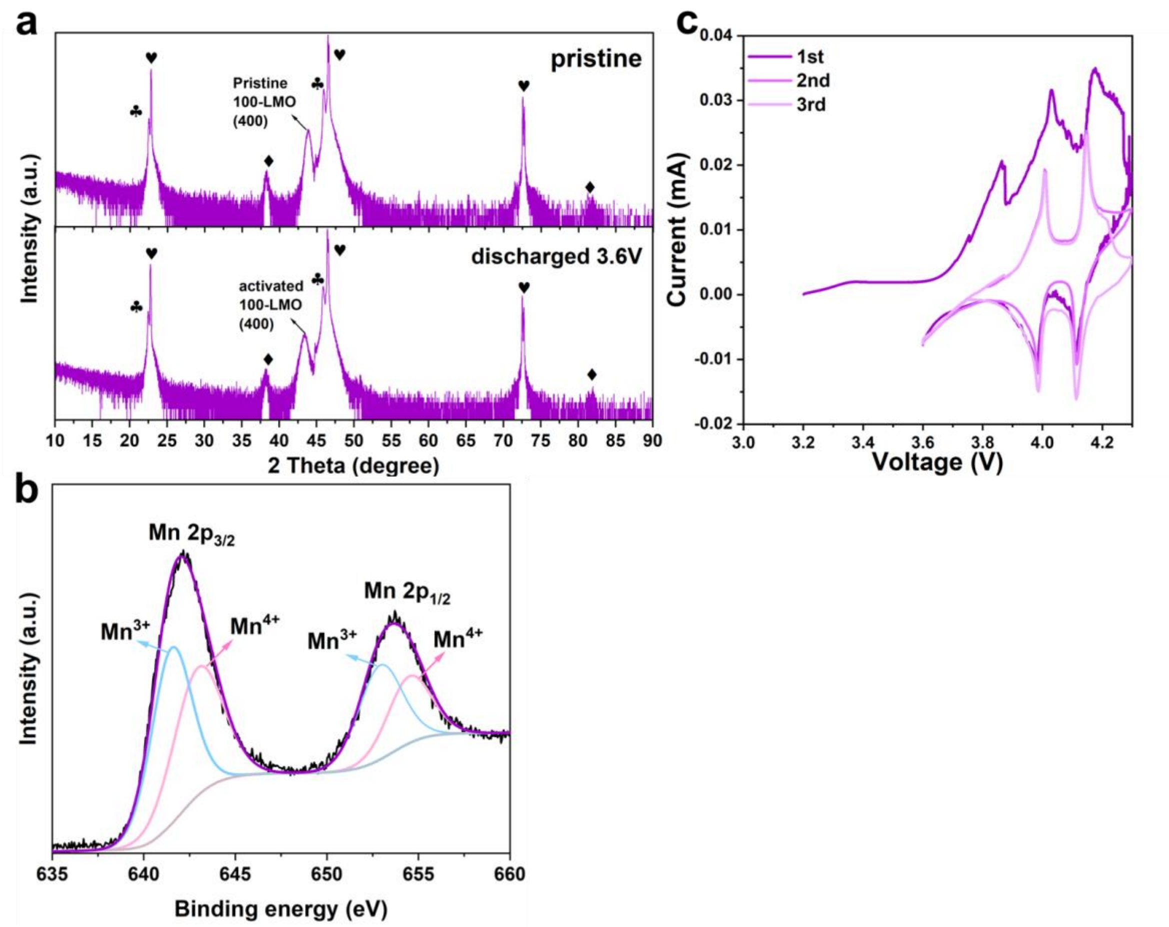

Figure S1. (a) XRD patterns of pristine 100-LMO and activated 100-LMO films (discharged 3.6 V); (b) Mn 2p XPS spectra of pristine 100-LMO film; (c) Initial three CV curves of activation process for 100-LMO film. Peaks from Nb-$SrTiO_3$ and $SrRuO_3$ are marked with ♥ and ♣, respectively. ♦ identifies the minor contribution of Mn_2O_3 impurity phase. The y-axis in (a) is log-scale.

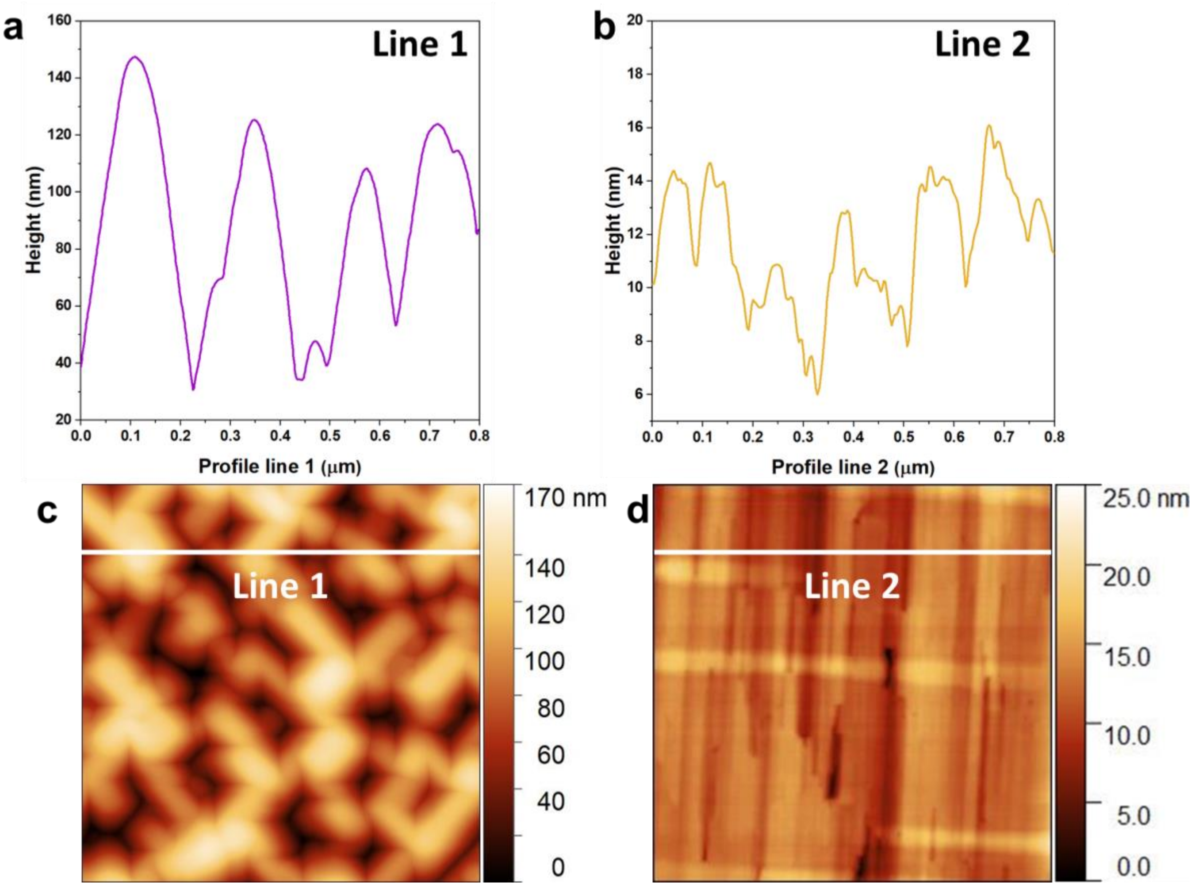

Figure S2. Surface height line profiles of (a) 100-LMO and (b) 110-LMO films; The corresponding AFM images of (c) 100-LMO and (d) 110-LMO films.

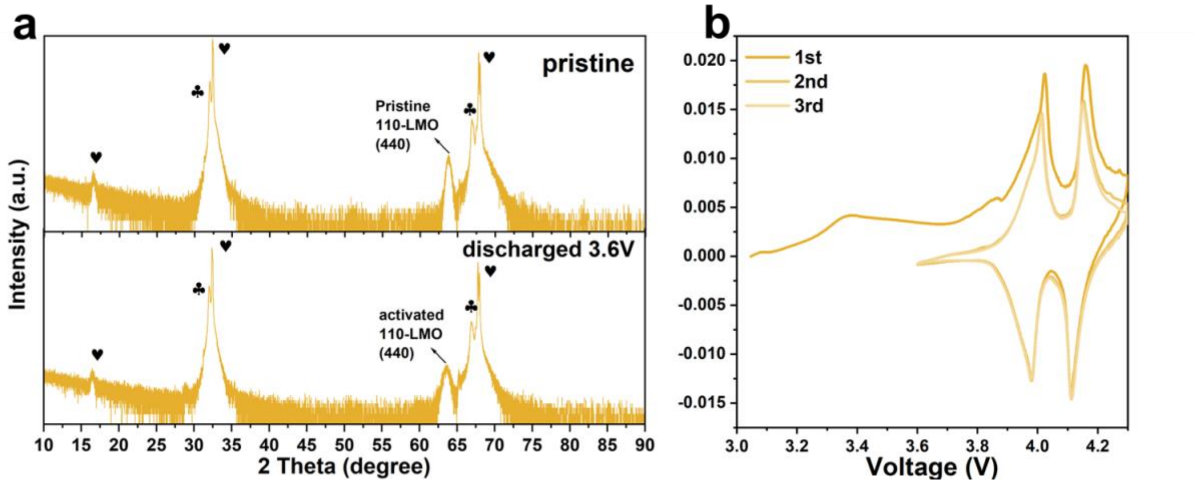

Figure S3. (a) XRD patterns of pristine 110-LMO and activated 110-LMO films (discharged 3.6 V); (b) Initial three CV curves of activation process for 110-LMO film. Peaks from Nb-$SrTiO_3$ and $SrRuO_3$ are marked with ♥ and ♣, respectively. The y-axis in (a) is log-scale.

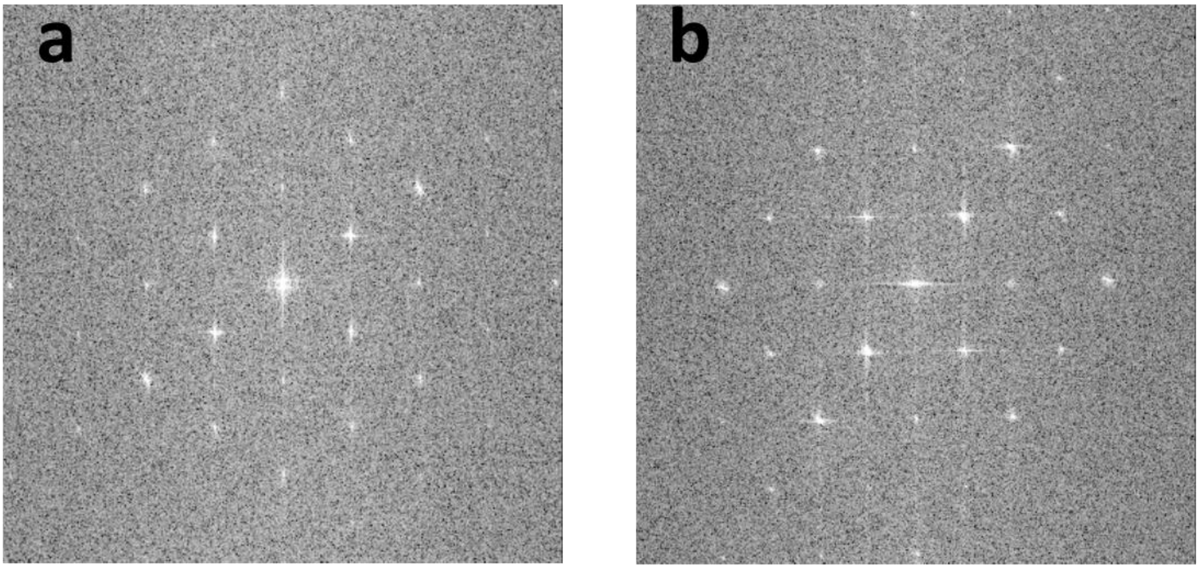

Figure S4. The raw FFT images of (a) 100-LMO film and (b) 110-LMO film that correspond to the yellow box areas in Figure 2a and 2c.

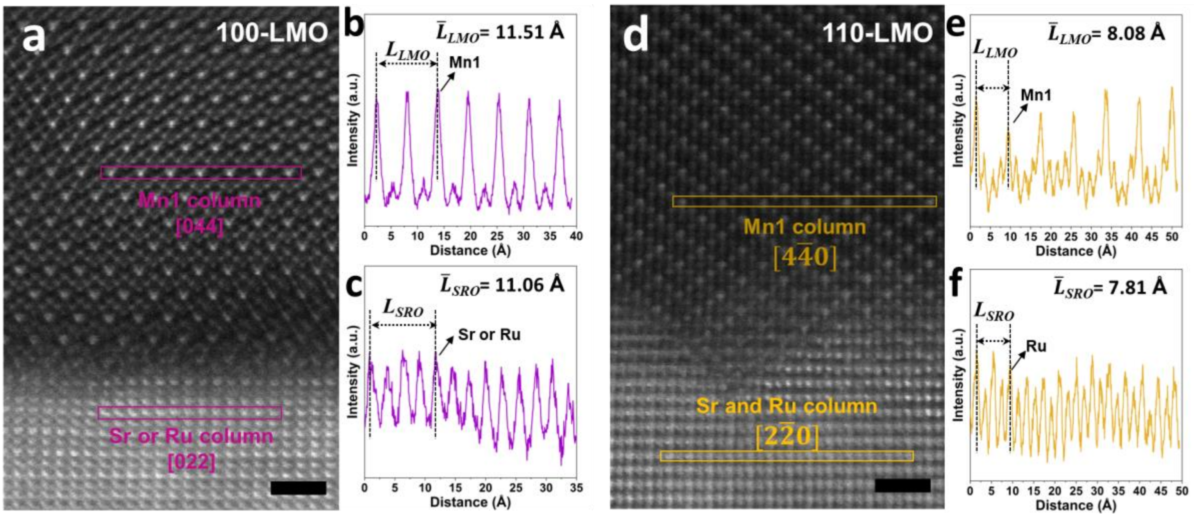

Figure S5. HAADF-STEM images of the (a) 100-LMO/SRO and (d) 110-LMO/SRO heterointerfaces; Line profiles of the (b) Mn1 column and the (c) Sr or Ru column along [011] direction in (a); Line profiles of the (e) Mn1 column and (f) Sr and Ru column along $[1\bar{1}0]$ direction in (d). Length of the scale bar: 1 nm.

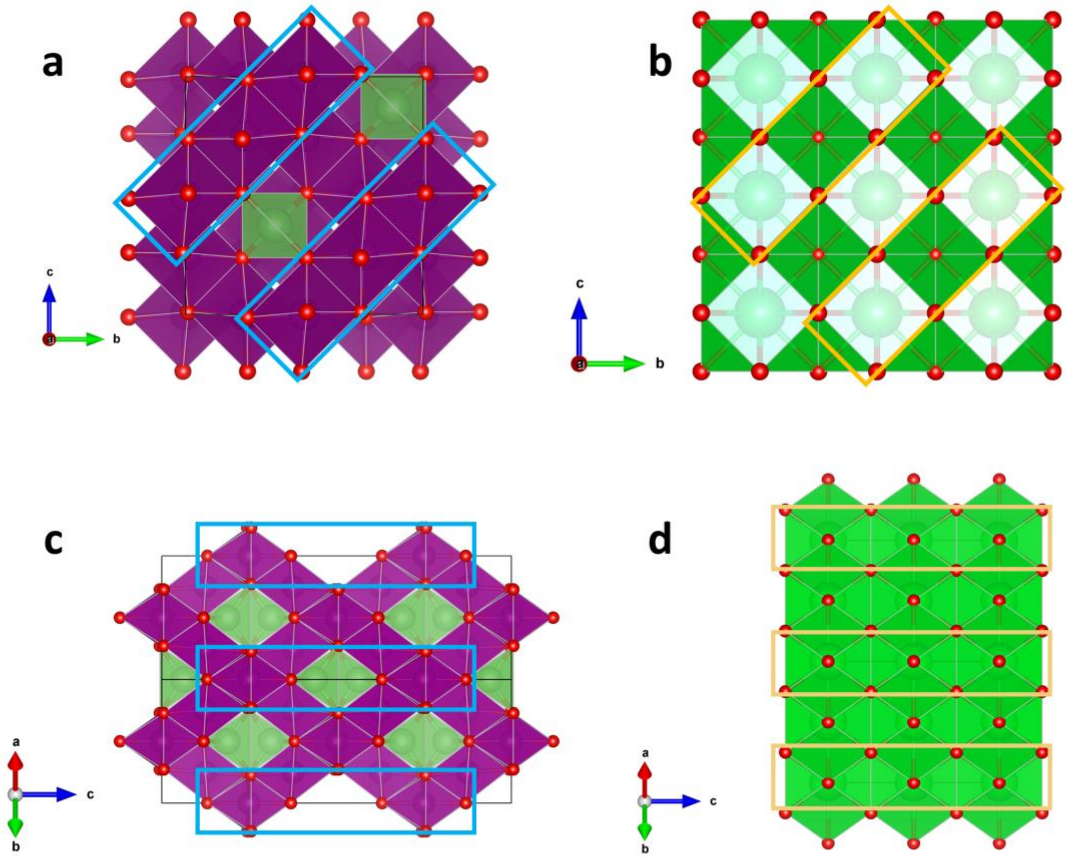

Figure S6. The schematic crystal structures of (a) LMO and (b) SRO along [100] zone axis; crystal structure of (c) LMO and (d) SRO along [110] zone axis. Blue and yellow boxes indicate the Mn columns and Sr columns, respectively.

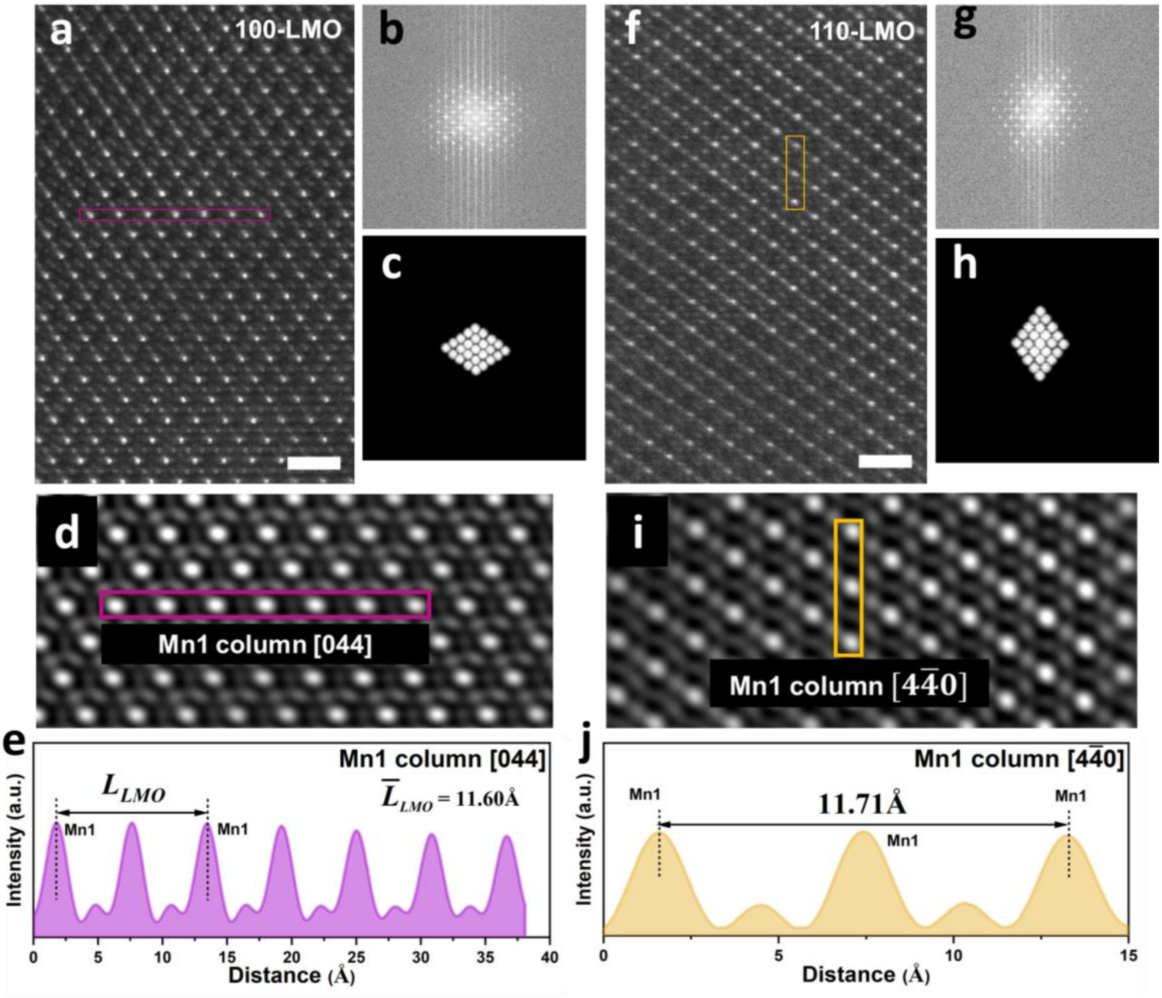

Figure S7. HAADF-STEM images and corresponding FFT images of the (a, b) 100-LMO and (f, g) 110-LMO film interior; Mask applied FFT images of (c) 100-LMO and (h) 110-LMO. Inverse mask applied FFT of (d) 100-LMO and (i) 110-LMO; Line profiles of the (e) Mn1 column along [011] direction in (d), and (j) Mn1 column along [1$\bar{1}$0] direction in (i). Length of the scale bar: 1 nm. Columns marked in (d) and (i) are the same as those marked in (a) and (f), respectively.

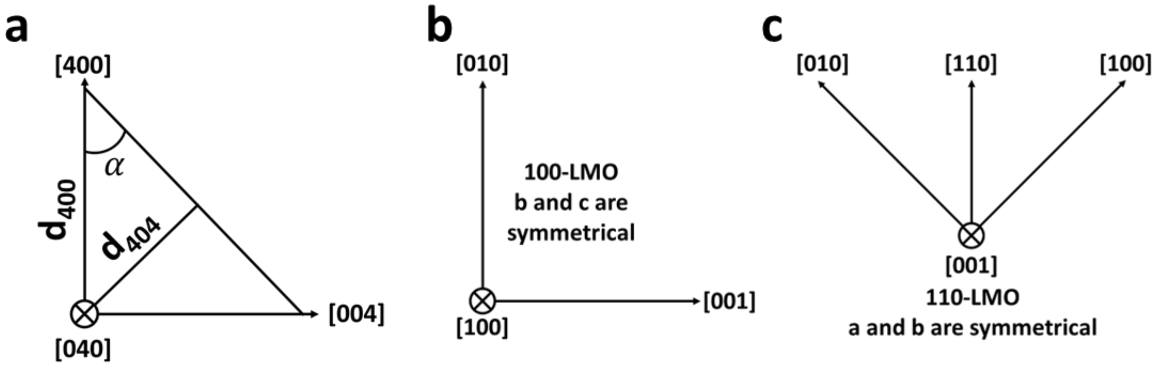

Figure S8. Schematic illustration of geometry relations in 100-LMO and 110-LMO models.

The geometry relationship between the d-spacing of (400) and (404) planes of the LMO structure (d_{400} and d_{404}) is illustrated for a bulk LMO unit cell in Figure S8a. The measured d_{400} and d_{404} values, can be related to the in-plane axis by the following equations:

$$\alpha = \sin^{-1}\left(\frac{d_{404}}{d_{400}}\right) \tag{S1}$$

$$d_{004} = d_{400} \times \tan\alpha \tag{S2}$$

where α represents the angle between (004) and (404) planes of LMO unit cells. Given the fact that the in-plane crystal planes of the bulk spinel structure are symmetric, the distorted in-plane axes should be undergoing the same compressed strain. Therefore, the *b* and *c*-axes of 100-LMO films are symmetric along the [011] zone axis (Figure S8b) while the *a* and *b*-axes of 110-LMO films are symmetric along the [110] zone axis (Figure S8c).

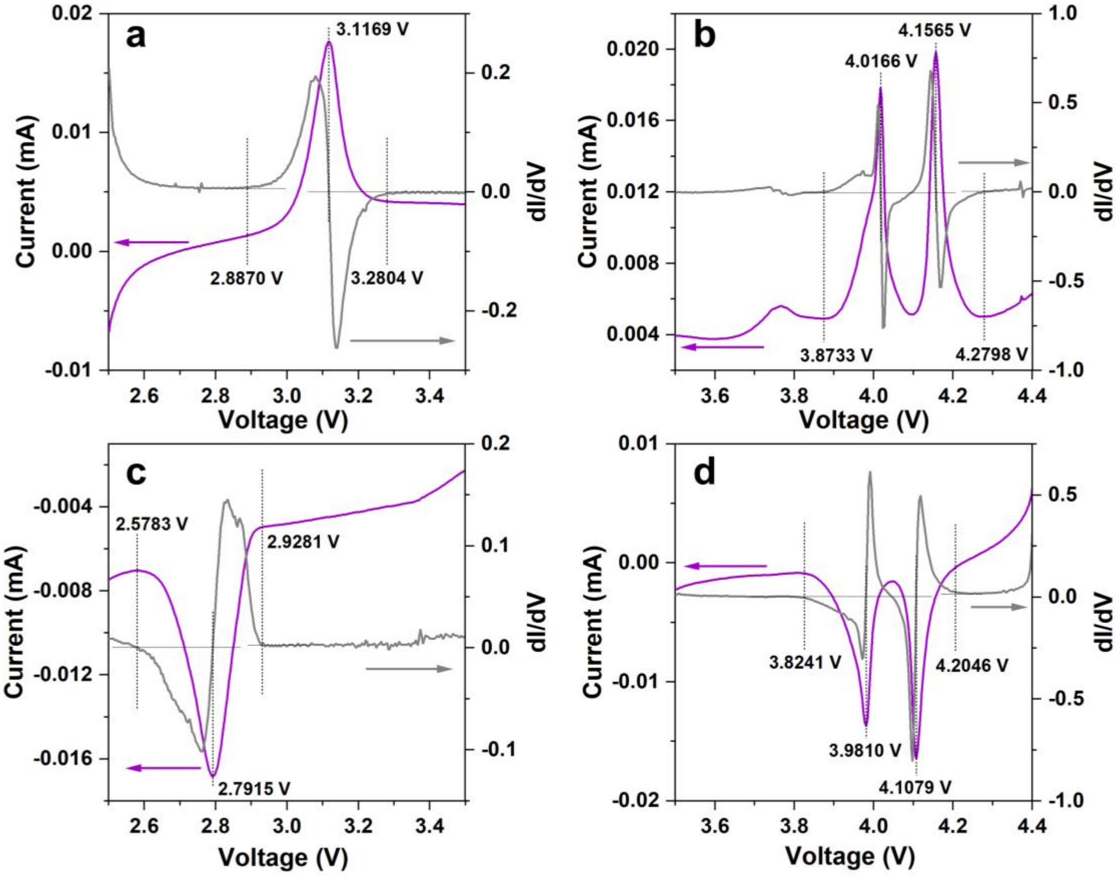

Figure S9. The oxidation peaks of a 100-LMO film at (a) 3 V range and (b) 4 V range; The reduction peaks of a 100-LMO film at (c) 3 V range and (d) 4 V range.

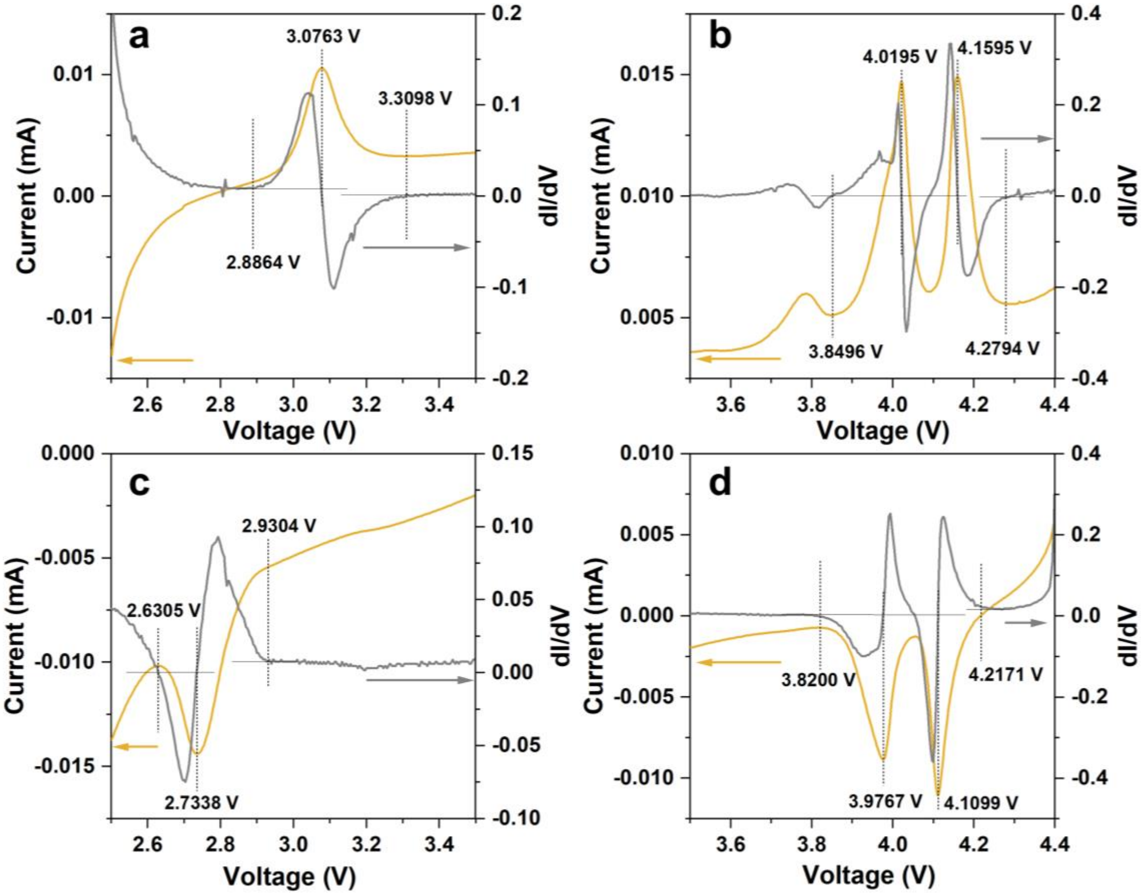

Figure S10. The oxidation peaks of a 110-LMO film at (a) 3 V range and (b) 4 V range; The reduction peaks of a 110-LMO film at (c) 3 V range and (d) 4 V range.

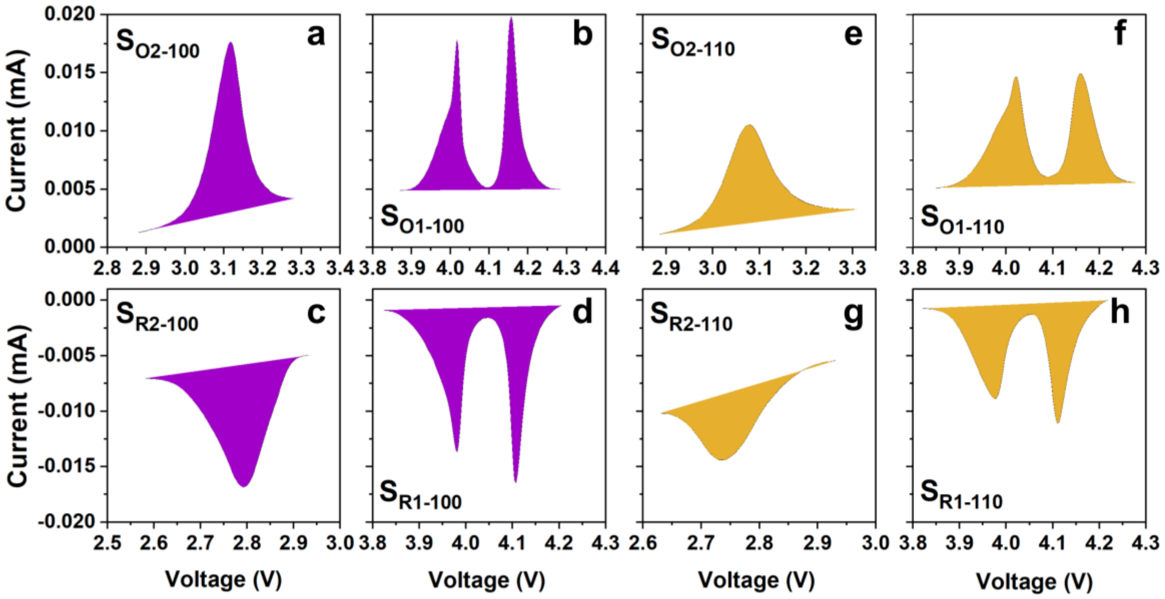

Figure S11. The defined areas of redox peaks for (a-d) 100-LMO and (e-h) 110-LMO films.

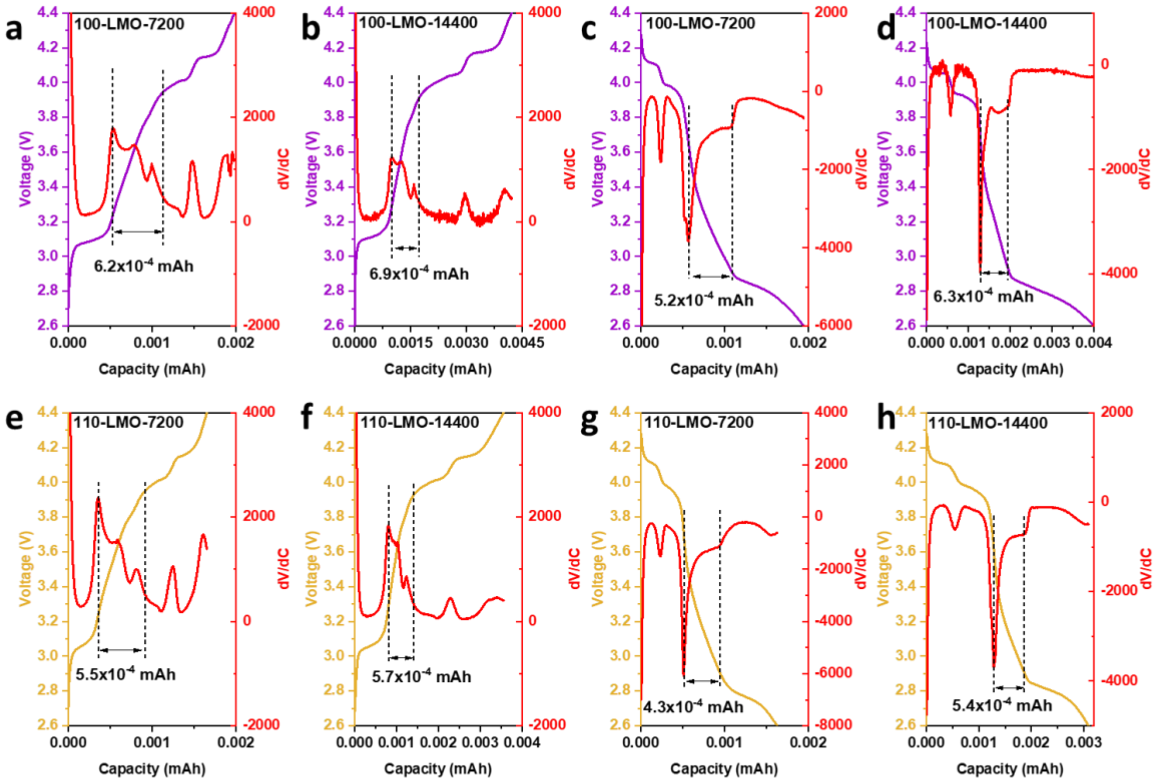

Figure S12. The charge/discharge profiles of (a-d) 100-LMO films and (e-f) 110-LMO films with different thicknesses. The applied current density for all films is 80 μA cm^{-2}. 7200 and 14400 indicate the applied pulses during the PLD deposition process.

Table S1. Summary of quantified capacity (presented by absolute in Coulombs) of the films in the 4V region, 3V region and middle region which are determined by first-order derivative curves shown in Figure S12. The capacities of additional 2nd and 3rd 100/110-LMO-14400 films are also shown to confirm that the measurements about capacity are statistically sound.

Sample	Quantified capacity (absolute in Coulombs, $\times 10^{-4}$ C)					
	Charge			Discharge		
	3 V<br>2.6-3.2V	Middle<br>3.2-3.9V	4 V<br>3.9-4.25V	3 V<br>2.95-2.75V	Middle<br>3.6-2.95V	4 V<br>4.25-3.6V
**100-LMO-7200**	1.48	1.72	2.02	1.56	1.44	1.52
1st 100-LMO-14400 (shown in Figure S12)	2.75	1.92	6.39	3.56	1.75	3.69
2nd 100-LMO-14400	3.08	2.01	5.14	3.96	1.57	3.08
3rd 100-LMO-14400	2.82	1.67	4.86	3.84	1.34	3.36
**110-LMO-7200**	0.97	1.53	1.89	1.42	1.19	1.11
1st 110-LMO-14400 (shown in Figure S12)	2.20	1.58	4.93	3.5	1.50	2.06
2nd 110-LMO-14400	2.08	2.22	4.90	3.22	1.74	2.10
3rd 110-LMO-14400	2.61	1.73	4.44	3.22	1.70	2.73

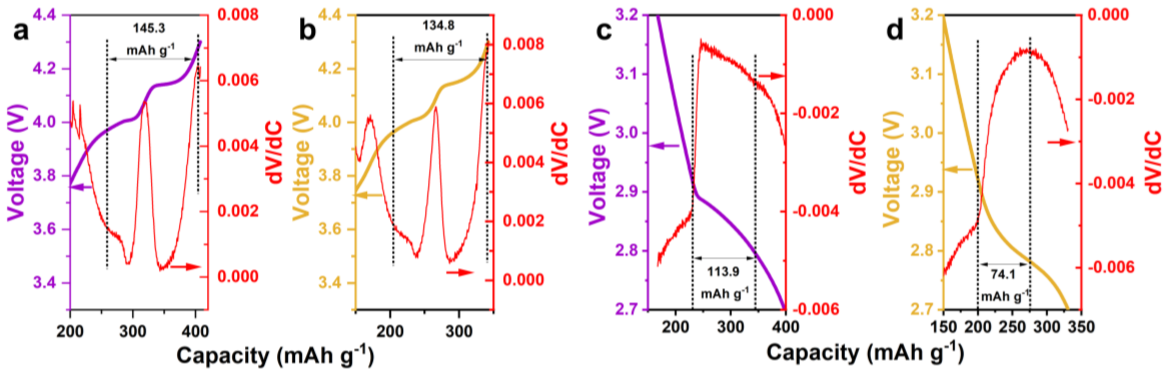

Figure S13. The charge/discharge curves and corresponding first-order derivative curves: 4 V plateau of (a) 100-LMO and (b) 110-LMO films; 3 V plateau of (c) 100-LMO and (d) 110-LMO films.

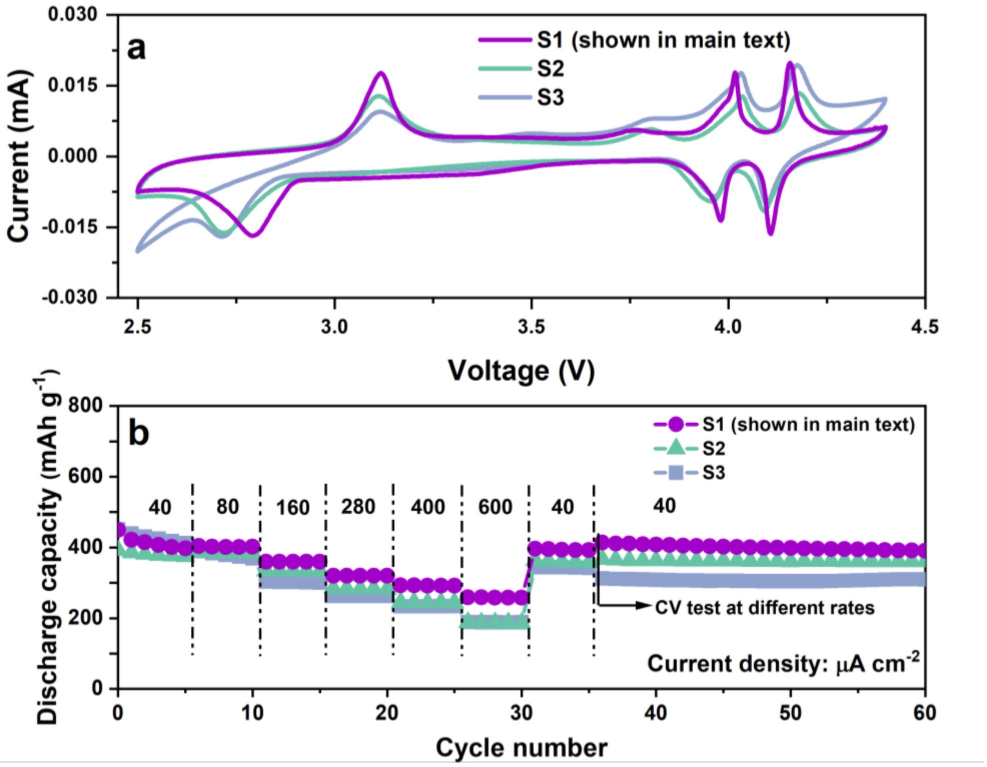

Figure S14: Reproducibility studies of 100-LMO films (S1, S2 and S3 indicated three individual 100-LMO film). (a) The CV curves at 1.0 mV s^{-1}; (b) The rate and cycling performance.

Although variations in CV curves and reversible capacities are observed and they are attributed to contact issues between Nb:STO substrates and electrochemical test devices (ECC-ref from EL-CELL), the reproducibility of good cycling stability of 100-LMO films is demonstrated.

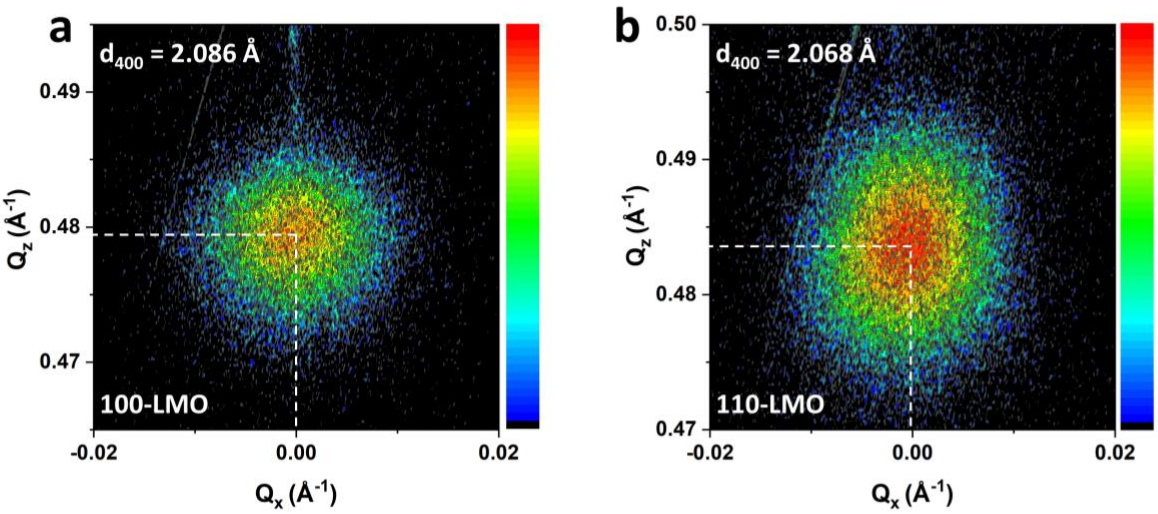

Figure S15. The magnified RSMs of (400) LMO peaks of (a) 100-LMO and (b) 110-LMO films after being charged from 2.5 to 3.6 V.

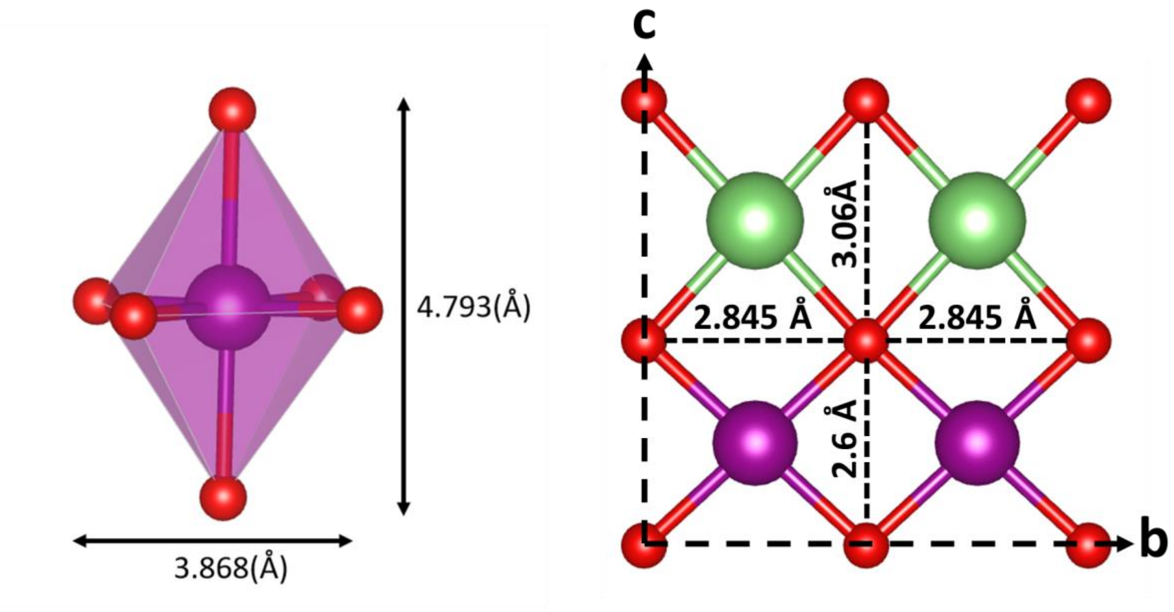

Figure S16. The average bond lengths of MnO_6 octahedra and $d_{O\text{-}O}$ values along *b* and *c* direction for bulk overlithiated $Li_2Mn_2O_4$ calculated by DFT modelling.

The DFT calculated lattice parameters for the bulk overlithiated $Li_2Mn_2O_4$ are b=c= 5.68 Å and a=9.45 Å. Due to a large anisotropy in Mn-O bond lengths of MnO_6 octahedra along the *bc* plane and *a* direction of the overlithiated phase, in which Mn^{3+} cations with large Jahn-Teller distortions are present, there exists a residual stress along b and c directions.

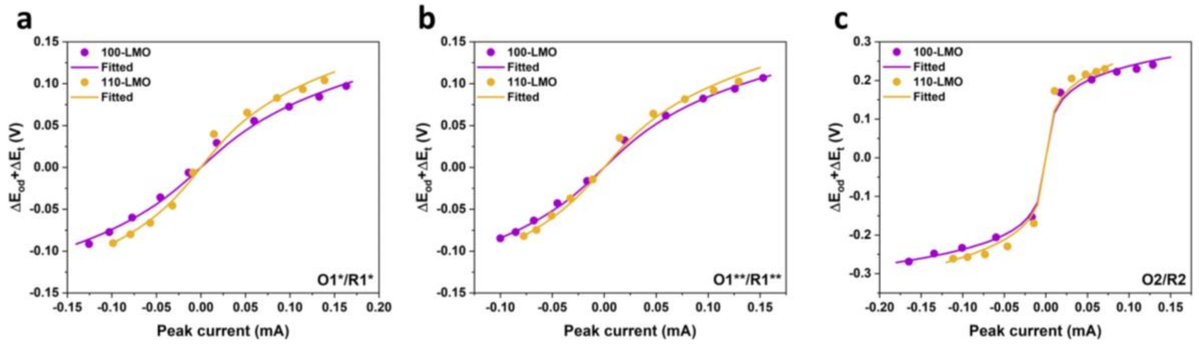

Figure S17. Fitted (ΔE_{od} + ΔE_t)-i_p lines for (a) O1*/R1*, (b) O1**/R1** and (c) O2/R2 redox peaks of 100-LMO and 110-LMO films based on equation (3);

Table S2. The fitted components of equation (3) for the 100-LMO and 110-LMO films.

Sample	Components					
	E_{eq} (V)		M		N	
	100-LMO	110-LMO	100-LMO	110-LMO	100-LMO	110-LMO
O1*/R1*	3.987	3.983	36001	50001	7.1822	3.8814
O1**/R1**	4.124	4.124	45004	53001	6.3131	9.9375
O2/R2	2.948	2.907	928210	1020000	0.4412	5.107
